# Different Derivatives of Plumbagin Analogue: Bioavailability and Their Toxicity Studies

**DOI:** 10.1002/fsn3.70720

**Published:** 2025-08-19

**Authors:** Souparnika Thekkumkara, Arenbenla Longchar, Baskar Venkidasamy, Benod Kumar Kondapavuluri, Muthu Thiruvengadam, Mansour Ghorbanpour, Sathianarayanan Sankaran

**Affiliations:** ^1^ Nitte (Deemed to be University), NGSM Institute of Pharmaceutical Sciences (NGSMIPS) Mangaluru Karnataka India; ^2^ Saveetha Dental College and Hospitals, Saveetha Institute of Medical and Technical Sciences Saveetha University Chennai India; ^3^ Dr. D.Y. Patil Medical College, Hospital and Research Centre Pimpri, Pune India; ^4^ Department of Applied Bioscience, College of Life and Environmental Science Konkuk University Seoul Republic of Korea; ^5^ Centre for Research Impact & Outcome Chitkara University Rajpura Punjab India; ^6^ Arak University Arak Iran

**Keywords:** bio‐availability, pharmacological action, plumbagin, plumbagin derivatives, toxicity

## Abstract

The roots of the *Plumbago* genus, specifically *Plumbago zeylanica* and related species, contain natural plumbagin. Because of its possible medicinal benefits, naphthoquinone has been the subject of several studies. Ongoing research aims to better understand the mechanisms underlying the actions of plumbagin, optimize its distribution and formulation, assess its efficacy and safety in clinical trials. Plumbagin is a bioactive compound that has several possible therapeutic uses; however, further studies are required to completely understand its advantages and disadvantages in clinical settings. The roots, leaves, and stem of the plant contain plumbagin, which possesses antimalarial, antiobese, antidiabetic, antimicrobial, antiulcer, anti‐inflammatory, antioxidant, and anticancer properties, and has been traditionally used to treat various disorders, including dysmenorrhea, leprosy, anemia, rheumatic pain, colds, coughs, and arthritis. Plumbagin has low bioavailability owing to its poor solubility, metabolic instability, and efflux by transporters. Although plumbagin has promising therapeutic properties, it is also poisonous, which limits its clinical applications, cytotoxicity, organ toxicity, oxidative stress, and genotoxicity. Plumbagin and its derivatives have high therapeutic potential, particularly in oncology and antibacterial applications. However, issues such as bioavailability and toxicity must be addressed. Advances in drug delivery technologies, chemical changes, and combination therapy are interesting approaches for increasing the therapeutic viability of plumbagin and its derivatives. To completely comprehend their therapeutic potential and guarantee human safety, further clinical investigations are needed.

## Graphical abstract

Plumbagin, discovered in *Plumbago zeylanica* , has potential therapeutic effects such as anticancer, antimicrobial, and anti‐inflammatory characteristics. It has traditionally been used to treat anemia and rheumatic pain, but its therapeutic application is limited because of low bioavailability and toxicity risk factors. Chemical modifications and combination medicines are being studied to improve delivery, reduce toxicity, and increase efficacy. More research and clinical trials are required to verify its safety and therapeutic potential.

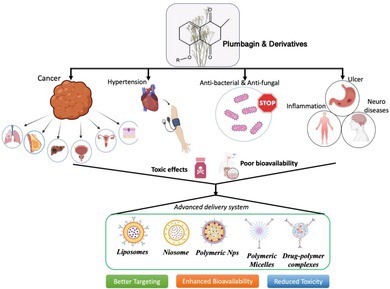

## 1 Introduction

A naphthoquinone, plumbagin (PB), is generated from the roots of various families of medicinal plants, including Droseraceae, Ebenaceae, and Plumbaginaceae. *Plumbago zeylanica* L. (Badwaik et al. 2019; Galal et al. 2013; Ganesan and Gani 2013) is often regarded as the best‐known medicinal plant that contains PB. PB has a very short half‐life; however, it is structurally identical to vitamin K. More significantly, PB has a limited oral bioavailability, mild toxicity, and low water solubility. Regardless, it appears to be a viable option for the development of new therapeutic medications. The biological activities of molecules, which include anti‐inflammatory, antifungal, antibacterial, antioxidant, and anticancer (Jiang et al. 2018; Yin et al. 2020; Titiwattanakarn et al. 2025) qualities, have been shown in numerous studies (Nazeem et al. 2009). The effects of PB on cancer have been thoroughly studied in the literature, and PB has promising therapeutic properties. It is also poisonous, which limits its clinical applications, cytotoxicity, organ toxicity, oxidative stress, and genotoxicity. PB and its derivatives have high therapeutic potential, particularly in oncology and antibacterial applications. However, issues such as bioavailability and toxicity must be addressed. Plumbagin, a natural substance with a variety of biological functions including anti‐inflammatory properties, is a good alternative for future medical research. Nevertheless, additional research is necessary to improve its clinical usefulness owing to its toxicity and absorption (Petrocelli et al. 2023). Advances in drug delivery technologies, chemical changes, and combination therapy are interesting approaches for increasing the therapeutic viability of PB and its derivatives. Plumbagin is a naphthoquinone with important pharmacological properties. Progress in the understanding of its biosynthesis and biotechnological manufacturing presents encouraging opportunities for long‐term and extensive medicinal applications. Although more research is required to address its pharmacokinetic and toxicity issues, plumbagin has varied therapeutic qualities, making it a fascinating prospect for future pharmaceutical research and development (Thakor and Janathia 2022). To guarantee human safety and complete comprehension of their therapeutic potential, further clinical investigations are needed. PB is a secondary metabolite found in several families, including Droseraceae, Drosophyllaceae, Ebenaceae, Nepenthaceae, and Plumbaginaceae. The primary component is 1,4‐napthoquinone. It is composed of a hydroxyl group at position five and a methyl substituent at position two. It contains a vivid yellow pigment. PB was disregarded in 1928 until Roy and Dutt looked into it; nonetheless, they were unable to derive the appropriate empirical formula. Madinaveitia and Gallego (1928) later elucidated the correct formula. The novelty of the review primarily provides a broad yet cohesive synopsis of the pharmacological potential of plumbagin. Plumbagin is a naphthoquinone produced by *P. zeylanica* and related species. It links modern pharmacological discoveries with various pathways to the traditional medicinal applications of plumbagin. This review emphasizes drawbacks, including toxicity, metabolic instability, and limited bioavailability. This highlights the need for improved delivery methods, chemical alterations, and improved clinical research. Further clinical investigations are needed to completely comprehend the therapeutic potential and guarantee human safety. Through the modulation of NF‐κB, PI3K/Akt, and STAT3, as well as ROS‐mediated mitochondrial pathways, plumbagin causes apoptosis and cell cycle arrest, and inhibits metastasis in a variety of cancer types, including breast, prostate, and lung cancer. It downregulates pro‐inflammatory cytokines and COX‐2 and NF‐κB signaling; therefore, it may be used as an anti‐inflammatory, antioxidant, and anti‐inflammatory agent that is beneficial in models of neurodegenerative diseases. Plumbagin shows potent activity against microorganisms, such as bacteria, fungi, and viruses. Plumbagin lowers glucose levels and protects heart tissue from ischemic damage in preclinical models. Plumbagin targets several important regulators including topoisomerases, Bcl‐2, STAT3, and p53. It is a multi‐target agent, which is a recent trend in contemporary drug research owing to its capacity to interact with several signaling cascades. To improve bioavailability, solubility, and target specificity, as well as address its intrinsic low water solubility and systemic toxicity, research is moving forward with nanoparticle‐based delivery, liposomal formulations, and prodrug designs. In cancer treatment, plumbagin works in concert with common chemotherapeutics (such as doxorubicin and cisplatin) to overcome drug resistance. Mechanistic research on immune response control, autophagy, and epigenetic modification is ongoing. Although it is currently in preclinical or early clinical stages, clinical translation is being studied. Owing to its broad‐spectrum biological activity, distinct mode of action, and intriguing therapeutic potential in areas where drug resistance or toxicity is an issue, plumbagin is a multipurpose phytochemical. Its uniqueness in contemporary biomedical research has been increased by continuous innovation in its formulation and molecular targeting.

## 2 Extraction of Plumbagin

The roots of *P. zeylanica* and *P. scandens* L. were believed to be the richest known sources of plumbagin(PB) when it was originally discovered. Grevenstuk et al. (2012) conducted the quantification analysis using n‐hexane as a solvent in a Soxhlet extraction process because it consistently produces a high yield, according to several studies. The PB in the plant roots, bark, leaves, aerial parts, etc., could not be quantified, even though the complete plant was used at the time of extraction. Despite its high yield, the Soxhlet method has several drawbacks. Specifically, the yield decreased as the extraction duration increased, showing that PB degraded after exposure to heat for longer than five hours. Therefore, to solve this issue, additional techniques for extraction were identified. As a result, other extraction techniques, such as hot and cold maceration (Sundari et al. 2017), were used to solve this issue. These results provide a full account of the extraction and isolation methods used in different studies (Badwaik et al. 2019). Tables 1 and 2 show the plumbagin spectroscopic and physical data, respectively.

**TABLE 1:** Plumbagin spectroscopic and physical data.

S. No	Biological source	Plant part	Green plant or dried powder	Method of extraction	Solvent used	References
1	*Dionea muscipula* Ellis	Whole plant	Dried powder	Soxhlet	Methanol	Kreher et al. (1990)
2	*Ceratostigma minus* Stapf Prain	Whole plant	Dried powder	Maceration room temp	Ethanol	Yue et al. (1994)
3	*Plumbago zeylanica* and other *Plumbago* spp.	Whole plant and their root	Dried powder of whole plant	Cold maceration	Toluene, glacial acetic acid and ethyl acetate	Hazra et al. (2004)
4	*P. zeylanica*	Root	Dried powder	Soxhlet	Methanol	Kishore et al. (2010)
5	*P. auriculata*	Root	Shaded dried powder	Soxhlet	Chloroform	Sreelatha et al. (2010)
6	*P. zeylanica*	Root	Dried powder	Cold maceration	Chloroform: dichloromethane (1:1)	Bothiraja et al. (2013)
7	*Drosera intermedia*	Whole plant	Dried powder	Soxhlet	n‐Hexane	Grevenstuk et al. (2012)
8	*P. zeylanica*	Root	Dried powder	Soxhlet	Methanol	Pradeepa et al. (2016)
9	*P. indica*	Root	Dried powder	Static extraction (maceration at roomtemperature)	Methanol and chloroform	Silja et al. (2016)
10	*P. indica*	Whole plant	Dried powder	Maceration (hot)	Ethanol	Makchuchit et al. (2017)
11	*D. anisandra*	Whole plant	Dried powder	Ultrasonication	n‐Hexane	Juárez‐Méndez et al. (2022)
12	*D. lusitanicum* L.	Leaves	Dried powder	Soxhlet and Ultrasonication	Methanol, chloroform and n‐Hexane	Grevenstuk et al. (2008)
13	*D. intermedia*	Whole plant	Dried powder	Ultrasonication and supercritical fluid extraction	Acetonitrile and water	Grevenstuk et al. (2012)
15	*P. europaea*	Aerial parts (leaves, flowers)	Dried powder	Clavenger	Water	Hasan et al. (2025)
16	*P. zeylanica*	Root	Dried powder	Microwave assisted extraction	Ethanol	Katoch et al. (2023)
17	*Juglans* spp.	Root, leaves	Dried powder	Supercritical fluid extraction	Carbon dioxide	Zenk et al. (1969)
18	*Triphyophyllum peltatum*	Stem, bark	Dried powder	Soxhlet extraction	Dichloromethane and 5% ammoniumhydroxide	Bringmann et al. (2008)

**TABLE 2:** Plumbagin physical and spectroscopic data.

S. No	Particulars	Data			References
1	Details about the molecular structure	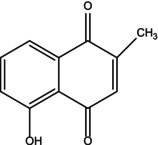 5‐hydroxy‐2‐methyl‐1,4‐napthaquinone			Bothiraja et al. (2011)
		Chemical formula: C_11_H_8_O_3_ Mol Wt: 188.182 g/mol, Elemental analysis: C:70.21, H:4.29, O:25.51. Orange to yellow needles (daylight), reddish, and M.P. 76°C–79°C Soluble in benzene, chloroform, ethyl alcohol, and acetone			
2	Spectroscopic interpretation	UV–Visible	UV: *λ*_max_ (methanol): 254 nm		Cesari et al. (2013)
			Visible: *λ*_max_ (methanol): 416 nm		
		IR	IR (KBr) peaks at 3321–3577 cm^−1^ for OH stretching, 1645–1667 cm^−1^ for carbonyl, 1609–1611 cm^−1^ for aromatic C]C, and 751.7–765 cm^−1^ for aromatic CH stretching		Bothiraja et al. (2011)
		NMR	^1^H NMR	^1^H NMR (CDCl3, 300 MHz): 2.19 (3H, s, Me‐2), 6.81 (1H, s, H‐3), 7.26 (1H, m, H‐6), 7.62 (2H, m, H‐7, 8) and 11.97 (–OH)	
				^1^H NMR (CD3OD, 500 MHz): 2.15 (3H, d, J¼ 1.6 Hz, CH3), 6.85 (1H, q, J¼ 2.0 Hz, H‐3), 7.24 (1H, d, J¼ 8.3 Hz, H6), 7.59. (1H, d, J¼ 7.4 Hz, H‐8), 7.65 (1H, dd, J¼ 8.3, 7.4 Hz, H‐7)	Cesari et al. (2013)
			^13^C NMR	^13^C NMR CDCl3 (75 MHz): 16.4 (2‐CH3), 114.9 (C‐4a), 119.1 (C‐8), 124.0 (C‐6), 131.8 (C‐8a), 135.3 (C‐3), 135.9 (C‐7), 149.5 (C‐2), 161.0 (C‐5), 184.6 (C‐1), and 190.1 (C‐4)	
				^13^C NMR CD3OD at 126 MHz: 192.5 (C‐4), 184.7 (C‐1), 161.1 (C‐5), 149.8 (C‐2), 136.1 (C‐7), 135.3 (C‐3), 132.4 (C‐9), 123.6 (C‐6), 118.8 (C‐8), 115.2 (C‐10), and 15.2 (C‐11)	Cesari et al. (2013)
		MS		EI‐MS: *m/z* (intensity%): 188 (100), 173 (26), 160 (27), 131 (44), 120 (30), 92 (39), and 63 (43)	Bothiraja et al. (2011)
				LC‐ ESI‐ MS: *m/z* 187.2 [M‐H]+	Singh et al. (2015)
3	Chromatography	TLC		Rf (n‐hexane: benzene (1:9)) = 0.64	Cesari et al. (2013)
				Rf (n‐hexane:ethylformate (9:1)): 0.37	Kapadia et al. (2005)
		HPTLC		Rf (mobile phase: n‐hexane: benzene (1:9), stationary phase: silica gel 60 F254 plates with a CAMAG LINOMAT IV automatic spotter): 0.63	Bothiraja et al. (2011)
4	Thermogram	DSC		Thermogram showed a significant melting endothermic peak at 78.6°C	Bothiraja et al. (2011)

### 2.1 Optimized Extraction Methods for Plumbagin

#### 2.1.1 Extraction With Microwave Assistance (MAE)

Using microwave energy to heat solvents that come into contact with plant materials, MAE effectively extracts phytochemicals (Katoch et al. 2023). Higher yields and shorter extraction times than traditional methods are the outcomes of this method's optimization for plumbagin extraction. The procedure is safe for the environment and appropriate for large‐scale operations.

#### 2.1.2 Extraction of Gelucire Without Solvent

Using the lipid‐based excipient Gelucire, this solvent‐free technique improves plumbagin intestinal absorption and solubility (Bothiraja et al. 2012). This technique is a potential method for creating oral formulations because it increases not only extraction efficiency but also bioavailability.

#### 2.1.3 Chloroform/Dichloromethane Cold Maceration

**FIGURE 1:**
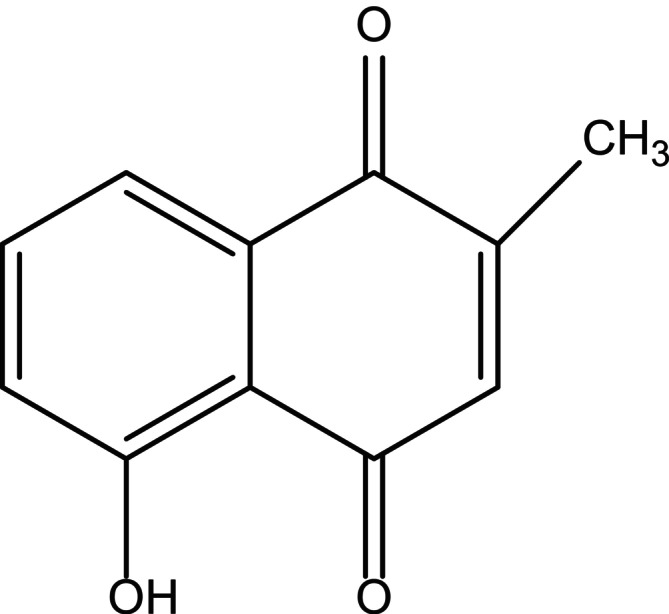
Structure of plumbagin.

Plumbagin is traditionally extracted by soaking the plant roots in a solution of chloroform and dichloromethane. Longer extraction durations and the use of hazardous solvents are significant disadvantages, despite the fact that it is efficient in producing pure chemicals (Bothiraja et al. 2011). Plumbagin structures have been studied using spectroscopic methods, such as FT‐IR, NMR, and MS (Figure 1). Plumbagin is classified as a hydroxy‐1,4‐naphthoquinone because it has methyl and hydroxy groups in place of hydrogen atoms at Positions 2 and 5. It functions as an antineoplastic agent, adjuvant for the immune system, anticoagulant, and metabolite. It is a hydroxy‐1,4‐naphthoquinone and a member of the phenol family (Ding et al. 2005).

## 3 Biological Activity of Plumbagin

Since it was found to be the main component of several naphthalene species, plumbagin(PB) has been linked to several pharmacological activities, including anticancer, neuroprotective, pro‐cognitive, anti‐Alzheimer's, antimicrobial, antidiabetic, analgesic, anti‐inflammatory, and anti‐arthritic effects.

### 3.1 Anticancer

**FIGURE 2:**
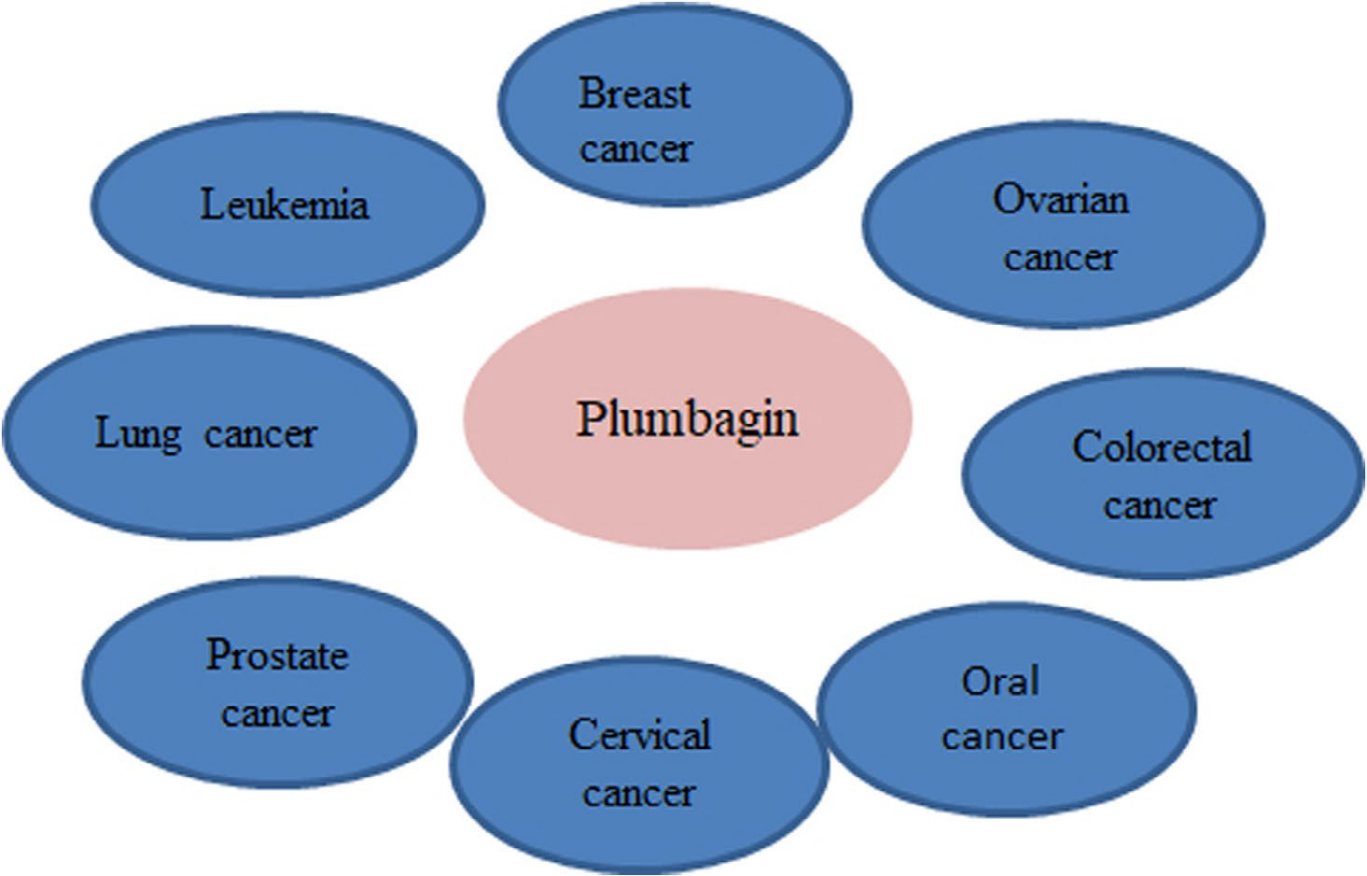
Therapeutic applications of plumbagin in various cancer types.

Cancer is a major cause of disease and mortality worldwide. According to WHO figures from 2018, cancer caused over 8.8 million deaths in 2015, accounting for nearly one in every six fatalities. Recent studies have predicted a significant increase in cancer cases worldwide. From 2022 to 2050, the number of cancer cases is predicted to increase by approximately 77%, reaching approximately 35.3 million cases globally (Bizuayehu et al. 2024). Low‐ and middle‐income nations are expected to see the largest increase in cancer incidence and death, and lung cancer continues to be the most prevalent and severe type of cancer in the world. Cancer incidence among those under 50 years of age has significantly increased, especially for cancers of the digestive system, which may be related to environmental and lifestyle factors. It is the world's second leading cause of death. Cancer is caused by various genetic and epigenetic changes that increase angiogenesis, metastasis, immune system malfunction, unregulated cell division, and resistance to apoptosis. In recent years, various treatments have been used to combat cancer evasion, including hormone therapy, surgery, radiation therapy, and chemotherapy. Chemotherapy is the most commonly used cancer treatment (Gomathinayagam et al. 2008). The development of medication resistance and substantial side effects are the primary factors that limit its effectiveness. As a result, many research teams have focused on evaluating the anticancer potential of various phytoconstituents, either alone or in combination with currently approved chemotherapeutic tablets. The potential of PB as an anticancer agent has been the subject of numerous studies. PB has been proven to have antineoplastic activity in both in vitro and in vivo tests of various cancer types (Badwaik et al. 2019). Figure 2 shows the use of plumbagin for various types of cancers.

#### 3.1.1 Breast Cancer

HIF‐1α translation is regulated by the PI3K/Akt/mTOR pathway. Previous studies have shown that it blocks the PI3K/Akt/mTOR signaling pathway in MCF‐7 cells, resulting in cell cycle arrest and autophagy (Xie et al. 2019). Suppressing the PI3K/Akt/mTOR signaling pathway reduces HIF‐1α synthesis in several cell types during hypoxia or CoCl_2_‐induced hypoxia, as shown in previous studies (Lee and Park 2011; Li et al. 2015; Yang et al. 2015). It also demonstrated that PB does not affect the expression of phosphorylated Akt, mTOR, or total PI3K under hypoxic conditions. The data suggest that PB, which inhibits the PI3K/Akt/mTOR signaling pathway in MCF‐7 breast cancer cells, is unlikely to interfere with HIF‐1α signaling or its target genes; however, PB may reduce HIF‐1α through an alternative mechanism. On the other hand, it may inhibit HIF‐1α by inhibiting the signaling pathway that is dependent on ERK and MPK. The various molecular processes and targets of HIF‐1α need to be further investigated to fully comprehend its inhibitory effect on PB. Furthermore, it has been shown that MCF‐7 cell lines express significant amounts of HIF‐1α protein (Shi et al. 2010). In numerous investigations, PB showed a potent anticancer effect on cell lines expressing the estrogen receptor (ER). Additionally, PB specifically targeted ERs that were highly expressed in MCF‐7 cells, while the opposite was true for MDA‐MB‐231 cells (Kilcar et al. 2016). This study aimed to investigate the effect of PB on HIF‐1α‐associated ER‐positive MCF‐7 cells. The impact of PB on ER‐negative MDA‐MB‐231 cells, on the other hand, is an intriguing topic that warrants more investigation.

#### 3.1.2 Prostate Cancer

Prostate cancer is a severe disease that affects millions of people and is the second leading cause of cancer‐related deaths among males in the United States. Hormone‐refractory prostate cancer is particularly aggressive, difficult to treat, and accounts for the majority of prostate cancer‐related deaths. According to Aziz et al. (2008), PB can also prevent the invasive growth of prostate cancer cells. PB only causes cancer cells to undergo apoptosis, which is in line with our findings in BRCA cells (Ahmad et al. 2008). The nontumorigenic prostate epithelial cell line, RWPE‐1, was unaffected by PB. Furthermore, PB (2 mg/kg body weight) delayed tumor growth in mice treated with DU145 cells for three weeks. Additionally, it reduced the tumor weight by 90%. It is interesting to note that the tumor did not develop more quickly for four weeks, even after quitting PB. This evidence suggests that PB suppresses hormone‐refractory prostate cancer cells. Furthermore, it effectively inhibited prostate cancer under both in vitro and in vivo conditions. (Powolny and Singh 2008) discovered that exposure to PB can significantly reduce the viability of prostate cancer cells with different receptor status. Additionally, PB treatment promoted apoptosis, which was linked to the formation of ROS. N‐acetylcysteine (NAC) pre‐treatment of cells inhibited PB‐mediated ROS formation and apoptosis, indicating that ROS production is an important factor in PB‐induced apoptosis.

#### 3.1.3 Ovarian Cancer

Thasni et al. (2008) examined how different chemical capacities to suppress cell development in ER‐positive ovarian cancer cells (Powolny and Singh 2008) depended on the BRCA1 (early onset) status of the cells. Plumbagin was the most effective of the chemicals studied. It induces apoptosis by attaching to and altering ER‐α in cells that have BRCA1 silenced. The cytotoxicity generated by PB was reduced upon silencing ER‐α. The chemotherapeutic potential of PB against ER‐positive tumors (Thasni et al. 2008), which are deficient or have mutated BRCA1, was suggested in this report. The impact of tamoxifen, emodin, and PB on ER‐expressing (Srinivas, Annab, et al. 2004) and BRCA1‐silenced ovarian cancer cells was studied by Srinivas. The ratio was PB 4 tamoxifen 4 emodin; apoptosis induction was more effective in cells prevented by BRCA1. BRCA1‐blocked cells were nearly twice as susceptible to PB treatment as control cells, requiring a dosage of 2.68 mM to kill 50% of the cells. Accordingly, five milligrams of PB was required to destroy half of the BRCA1‐blocked cells, suggesting that PB may be a helpful therapeutic intervention for cancers caused by BRCA1 mutations.

#### 3.1.4 Myeloma

The known benefit of activating STAT‐3 is that it can slow down the development of cancer. Therefore, the inhibiting of STAT‐3 activation may be essential to stop the spread of the disease. Sandur et al. (2010) investigated the modulation of PB STAT‐3. Constitutively active STAT‐3 inhibited both constitutive and interleukin (IL)‐6‐inducible STAT‐3 phosphorylation, and this suppression was effective in plumbagin‐induced apoptosis. This study used the human multiple myeloma cell lines MM.1S and U266 to demonstrate that PB has a favorable effect. A prior study (Bringmann et al. 2008), which examined the proliferation of the human multiple myeloma cell lines RPMI8226 and INA‐6 using Annexin V‐FITC/PI labeling, found no indication of PB impact. Dioncoquinone A and Dioncoquinone B, two naturally occurring naphthoquinones isolated from *Triphyophyllum peltatum*, were found to be effective against myeloma cell lines. Two naturally occurring naphthoquinones, dioncoquinone A and dioncoquinone B, isolated from *T. peltatum* , were found to be effective against myeloma cell lines. According to these findings, PB may influence myeloma models in different ways, depending on the cell source. Further research is needed to fully understand the anticancer activity of PB in multiple myeloma.

#### 3.1.5 Pancreating Cancer

According to Chen et al. (2009), plumbagin possesses anticancer properties against pancreatic cancer and has the lowest chance of survival. The chemical had a dose‐dependent effect on the growth of Panc‐1 and BxPC‐3 pancreatic cancer cells. Plumbagin‐treated Panc‐1 cells showed morphological alterations that matched apoptosis, according to discoloration and transmission electron microscopy examinations. The number of apoptotic cells increased dramatically, and a variety of procedures have been utilized to assess apoptosis. Apoptosis‐inducing factor was overexpressed in the cytosol, mitochondrial transmembrane potential rapidly dropped, pro‐caspase‐9 and PARP (poly ADP‐ribose polymerase) were degraded, and Bax was increased following PB exposure. PB anticancer effects against prostate cancer have been studied by Chen and associates. It was shown that the fluorometric substrate assay activated caspase‐3 but not caspase‐8. In an orthotopic pancreatic tumor model, PB significantly reduced Panc‐1 xenograft development but had no discernible effect on body weight or leukocyte counts. Therefore, PB may be developed as a new treatment option for pancreatic cancer (Chen et al. 2009).

#### 3.1.6 Lung Cancer

Currently, there is limited therapeutic success with various treatments for lung cancer. The cause of this is that non‐small‐cell lung cancer (NSCLC) develops resistance to regular treatment. PB dramatically reduced the proliferation of H460 cells compared to A549 cells, according to Gomathinayagam et al. (2008), who investigated the anticancer activities of PB against H460 and A549 NSCLC. EGFR‐mediated signaling was downregulated in these cells by 80% PB. Furthermore, activating caspase‐3 to induce apoptosis (Hsu et al. 2006) and altering a number of cell cycle regulatory factors results in cell cycle arrest. PB has also been demonstrated to inhibit cell proliferation by inducing A549 cell death and cell cycle arrest and has been shown to alter the expression of numerous cell cycle‐regulating proteins, including p21, cyclin B1, Cdc2, Cdc25C, and others. Moreover, PB administration was found to trigger the mitochondrial apoptotic pathway, and a model employing naked mice further validated the anticancer impact. For PB, Acharya et al. (2008) evaluated its impact on cellular microtubules. Purified tubulin polymerization was effectively stopped by plumbagin, which also disrupted the interphase microtubule network in a dose‐dependent manner. The possible cause of the reported effects of PB was hypothesized to be the identification of the colchicine binding site of tubulin. Taken together, these results suggest that PB can be used to treat lung cancer as an anticancer drug. Further clinical research is needed to determine the therapeutic potential of this intriguing natural substance. PB was discovered to be an effective inhibitor of TPA‐induced invasion and migration in A549 cancer cells (Shieh et al. 2010). PB's ability to decrease MMP and urokinase‐type plasminogen activator (uPA) expression has been connected to this activity.

#### 3.1.7 Skin Carcinoma

Plumbagin has been shown to have anticancer properties in human melanoma cells (Wang et al. 2008). PB suppressed the growth of A375 cells by causing S‐G2/M cell cycle arrest and death of S2 cells. PB has the ability to prevent cancer and has been established in vivo. During PB therapy, cyclin B1, A, Cdc2, and Cdc25C levels decreased, while p21 levels increased. Activating caspase‐9 and changing Bax/Bcl‐2 ratio also caused apoptotic cell death. Additionally, PB suppresses cell growth (Nazeem et al. 2009). Used A‐431 cells to examine the biological function of plumbagin in the redox recycling of copper, a transition metal, by employing ROS scavengers and neocuproine, a Cu(I)‐specific chelating agent, has demonstrated that PB anticancer actions are mediated via ROS and Cu(I). PB inhibits proliferation and induces death in SKBR3 BRCA cells, indicating its anticancer properties. Other researchers have also demonstrated the anticancer activity of PB against melanoma cells (Prasad et al. 1996; Mandala Rayabandla et al. 2010).

#### 3.1.8 Liver Cancer

Plumbagin has been shown to reduce MMP‐2 and uPA (Jaiswal et al. 2002; Shih et al. 2009) levels, preventing the migration and infiltration of HepG2 cells in liver cancer cells, it leads toinhibiting the growing liver cancer cells p300, located in close proximity to lysine acetyltransferase, is associated with numerous diseases due to its dysfunction, and it controls multiple genes. PB can inhibit p300‐mediated p53 acetylation and histone acetyltransferase activity in vivo (Ravindra et al. 2009). PB, by inhibiting p300 histone acetyltransferase activity, may affect a number of genes associated with illnesses, such as cancer. The hydroxyl group of PB forms a hydrogen bond with the lysine 1358 residue of p300, according to the study. This functional group is important because PB derivatives with altered or replaced C‐5 hydroxyl groups lose their ability to inhibit acetyltransferase. PB administration increases gluconeogenic enzyme levels, indicating a mechanism for the anticarcinogenic effects of 3‐methyl‐4‐dimethylaminoazobenzene (3MeDAB) ‐induced hepatoma (Parimala and Sachdanandam 1993). Forty Wistar male rats with 3MeDAB‐induced hepatoma, PB (4 mg/kg body weight), were found to dramatically inhibit tumor growth. PB was previously found to be cytotoxic to the hepatoma cell line HEPA‐3B (Kuo et al. 1997).

#### 3.1.9 Renal Cancer

NAD(P)H oxidase Nox‐4 is found in the kidney and is predominantly expressed in brain tumor cells (LN229) and human embryonic kidney 293 (HEK293) cells. Ding et al. (2005) used lucigenin‐dependent chemiluminescence to investigate the effect of plumbagin (PB) on Nox‐4 activity in HEK293 cells. This study also used LN229 brain tumor cells. PB suppressed Nox‐4 activity in HEK293 and LN229 cells in a dose‐ and time‐dependent manner. The superoxide inhibitor diphenyleneiodonium, which inhibits NADPH oxidase, inhibited HEK293 cells. HEK293 cells required both NADPH and NADH to produce superoxide, demonstrating that NADPH oxidase in these cells produced superoxide, but not the lucigenin redox cycle. PB also inhibited Nox‐4's superoxide generation. The COS‐7 cells were then transfected. PB has shown that PB directly blocks Nox‐4 activity in several investigations.

#### 3.1.10 Cervical Cancer

To treat cervical carcinoma, Kuo et al. (1997) used the HeLa cell line to establish plumbagin's (PB) anticancer properties. Srinivas, Gopinath, et al. (2004) investigated the efficacy of PB treatment in human cervical cancer cells, ME 180. Their data indicate that the PB was effective suppressors of cell division. PB therapy induces apoptosis by activating caspase‐3 and caspase‐9, phosphatidylserine migration, nuclear condensation, and DNA fragmentation. A fluorescence test was performed to determine whether PB and its quinone derivatives were cytotoxic to HeLa cells (Montoya et al. 2004). Apoptosis was identified by morphological changes caused by these chemicals, including blebbing and nuclear condensation. PB can be employed with C33A cells because it has been demonstrated to cause apoptosis in these cells and has been demonstrated to cause C33A (Nair et al. 2008) cells to undergo apoptosis. When paired with modest radiation dosages, it efficiently inhibits cell proliferation without requiring high radiation dosages.

#### 3.1.11 Inhibition of NF‐kB Activity

Plumbagin significantly reduces NF‐kB activation caused by various stressors (Sandur et al. 2010). PB inhibits constitutive NF‐kB in cancer cells, which inhibits downstream NF‐κB‐regulated gene products, as demonstrated in this study. These findings suggest that PB may have radiosensitizing, cell growth‐modifying, and anticarcinogenic effects. Our findings revealed dose‐dependent inhibition of endogenous NF‐kB when PB levels in BRCA cells were increased. This effect was observed in both ER‐negative MDA‐MB‐231 and ER‐positive MCF‐7 cells, suggesting a way to eliminate both cell types. PB inhibits the NF‐κB/Bcl‐2 pathway, which causes apoptosis in BRCA cells. Suppression of the NF‐kB/Bcl‐2 pathway by PB could be clinically significant for treating advanced and refractory BRCAs, as NF‐kB activity and Bcl‐2 expression are associated with chemotherapy resistance (Buchholz et al. 2005). Table 3 shows the anticancer activities of the action of plumbagin.

**TABLE 3:** Anticancer activities of plumbagin with mechanism of action.

S. No	Types of cancer	Research models	Pathways/mechanism affected by plumbagin	References
1	Leukemia	APL cell line NB4	Modulation of mitochondrial pathway	Zhao and Lu (2006)
		NOD/SCID mice with NB4 tumor xenograft	Formation of ROS	Sun and McKallip (2011)
		K562 human chronic myelogenous leukemia cells	Formation of ROS and increased expression of TRAIL death receptor	Bae et al. (2016)
		T‐cell acute lymphoblastic leukemia MOLT‐4 cells	Augmentation of caspase‐dependent apoptosis	Bae et al. (2016)
		CLL cells	Reduction of Bcl‐2 and increase in the level of Bax protein	Uttarkar et al. (2016)
		AML cells	Inhibition of c‐Myb binding to coactivator p300	Uttarkar et al. (2016)
		HL‐60 cells	Formation of ROS and selective inhibition of thioredoxin reductase	Zhang et al. (2017)
		HLK‐1 cells	Increased expression of TRAIL death receptor, activation of caspase‐8 and inhibition of cFLIP	Kong et al. (2017)
2	Breast cancer	MDA‐MB‐231 and MCF‐7 cells	Inhibition of Akt/mammalian target of rapamycin pathway, inactivation of Bcl‐2 and NF‐κB and arrest of G2‐M cell cycle	Kuo et al. (2006) and Ahmad et al. (2008)
		MDA‐MB‐231 and BT474 cells	Decreased expression of chemokine receptor XCR4	Kawiak et al. (2012)
		SKBR3 and BT474 cells	Decreased levels of anti‐apoptotic Bcl‐2 and increased levels of pro‐apoptotic Bcl‐2 family of Proteins	Yan et al. (2014)
		MDA‐MB‐231SA cells	Reduced bone erosion area, number of TRAcP positive osteoclasts and prevented decrease in bone tissue volume	Sung et al. (2012)
		Mice with human breast cancer‐induced osteolytic bone metastasis	Abrogated the RANKL triggered NF‐κB and MAPK signaling pathways by blocking association of RANK with TRAF6 in osteoclastogenesis	Lee et al. (2012) and Zhang et al. (2016)
		MCF‐7 cells	Upregulation of p53 and p21 mediated arrest of G1 cell cycle, and inhibition of PI‐5 kinase to generate ROS	Yan et al. (2013)
		Human breast cancer MDA‐MB‐231SArfp cells	Inhibition of Stat3 signaling and downregulation of TGF‐β, IL‐1α, MMP‐2 and MMP‐9	Somasundaram et al. (2016)
		BRCA1‐defective breast cancer cells	Formation of ROS to suppress plasticity of breast cancer stem cells	Nair et al. (2016)
		Homologous recombination defective triple negative BRCA1 mutant cells, and cancer bearing mice	Formation of ROS to trigger DNA double‐strand breaks	Kawiak et al. (2017)
		HER2‐overexpressing breast cancer cells	Inhibition of IKKα mediated activation of NF‐κB. MCF‐7 and T47D cells Inhibition of glucose regulated protein 78 and upregulation of Bik	Powolny and Singh (2008)
3	Prostate cancer	PC‐3, LNCaP, and C4‐2 cells	Formation of ROS and depletion of intracellular GSH levels	Lai et al. (2012)
		Prostate cancer xenograft mouse models	Blockade of Ras/Rac/cofilin and Ras/MEK signaling pathways mediated by VEGFR2 in HUVECs	Hafeez et al. (2012)
		Excised prostate tumor tissues	Inhibition of the expression of PKCε, Stat3, proliferating cell nuclear antigen and neuroendocrine markers (synaptophysin and chromogranin‐A)	Hafeez et al. (2013)
		PC‐3M‐luciferase cells in an orthotopic xenograft mouse model	Inhibition of the expression of PKCε, p Stat3Tyr705, and p Stat3Ser727, Stat3 downstream target genes survivin and Bcl(xL), proliferative markers Ki‐67 and PCNA, metastatic marker MMP9, MMP2, and uPA, and angiogenesis markers CD31 and VEGF	Hafeez et al. (2015)
		Primary and CRPC in PTEN‐KO mice	Inhibition of PKCε, Stat3, AKT, and EMT markers (vimentin and slug)	Qiu et al. (2015)
		PC‐3 and DU145 cells	Inhibition of epithelial‐to‐mesenchymal transition, and sirtuin1‐ and PI3K/Akt/mTOR‐mediated pathways	Zhou et al. (2015)
		Hormone‐refractory prostate cancer cells	Formation of ROS	Huang et al. (2018)
		PTEN‐P2 cells	Modulation of gene expression through androgen receptor	Son et al. (2010)
	Ovarian cancer	Suppress cell development in ER‐positive ovarian cancer cells depended on the BRCA1	Helpful therapeutic intervention for cancers caused by BRCA1 mutations	Thasni et al. (2008)
	Myeloma	Activating STAT‐3	Slow down the development of cancer	Sandur et al. (2010)
	Pancreatic cancer	Treated Panc‐1 cells	Significantly reduced Panc‐1 xenograft development	Chen et al. (2009)
	Lung cancer	Reduced the proliferation of H460 cells when compared to A549 cells	Ability to decrease MMP and urokinase‐type plasminogen activator (uPA) expression	Shieh et al. (2010) and Gomathinayagam et al. (2008)
	Skin carcinoma	Cell growth in A375 by causing S‐G2/M cell cycle arrest and death S2 cells	Inhibits proliferation and induces death in SKBR3 BRCA cells	Prasad et al. (1996) and Mandala Rayabandla et al. (2010)
	Liver cancer	Inhibit p300‐mediated p53 acetylation and histone acetyltransferase	Reduce MMP‐2 and uPAlevels, preventing liver cancer cells from migrating and infiltrating HepG2 cells	Jaiswal et al. (2002) and Shih et al. (2009)
	Renal cancer	NADPH and NADH to produce superoxide	Block Nox‐4 activity	Ding et al. (2005)
	Cervical cancer	HeLa cell line	Inhibits cell proliferation	Nair et al. (2008)

### 3.2 Antibacterial Activity of Plumbagin

**FIGURE 3:**
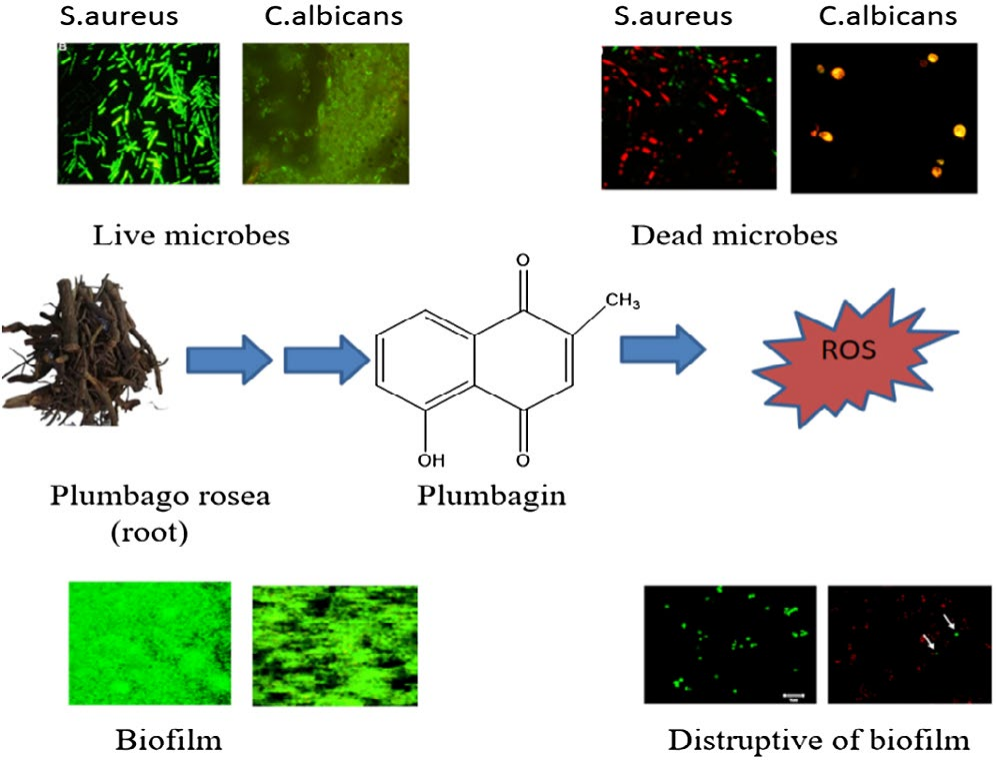
Antibacterial activity of Plumbagin.

Several studies have reported the antibacterial action of plumbagin. PB can effectively treat germs resistant to antibiotic therapy. The bacterium *Staphylococcus aureus* is a gram‐positive bacterium, and *Candida albicans* can co‐infect and cause sepsis, pneumonia, skin infections, urinary tract infections, and disorders linked to biofilms (Nair et al. 2008). PB exhibits significant antimicrobial activity against *C. albicans* and *S. aureus* (Rondevaldova et al. 2015). PB is effective in preventing Gram‐negative *Escherichia coli* and *S. aureus* from developing antibiotic resistance (Durga et al. 1990). Additionally, it improved the antibacterial efficacy of tetracycline and oxacillin against *S. aureus* strains that were resistant to these drugs. PB boosted the antifungal efficacy of amphotericin B against *C. albicans* . *Mycobacterium tuberculosis* (MTB) has been found to be resistant to both first‐ and second‐line anti‐tuberculosis medications. Plumbagin appeared to have good results. When it came to resistant *Helicobacter pylori* and Mycobacterium TB, PB showed encouraging results. By lowering polymerase activity and nucleoprotein production, *P. indica* extract suppresses the influenza A (H1N1) pdm09 virus (Hassan et al. 2016). Additionally, PB may have anti‐measles and anti‐hepatitis C virus effects, and in some areas, leishmaniasis, a disease transmitted by infected sandflies, poses a serious threat to public health. PB suppresses *Leishmania donovani, L* *. mexicana* , and *L. amazonensis*. Chagas disease is caused by the protozoan parasite *Trypanosoma cruzi,* which can cause major damage to digestive and cardiac systems. PB has been shown to have antitrypanosomal properties. PB has been found in vitro to be effective against both 3D7 chloroquine‐sensitive malaria parasites and K1 chloroquine‐resistant *Plasmodium falciparum* clones; when mice were infected with the *Plasmodium berghei* ANKA strain, PB showed antimalarial activity (Sumsakul et al. 2014). There is evidence that PB deters mosquitoes as well (Pradeepa et al. 2016) (Figure 3). The antibacterial and antiviral properties of plumbagin greatly enhance its therapeutic potential, particularly for drug‐resistant infections, chronic microbial illnesses, and new viral threats (Figure 3). Given its diverse modes of action and potential synergy with current medications, it is a promising candidate for further pharmacological development (Table 4).

**TABLE 4:** Plumbagin antimicrobial and antiviral activity.

S. No	Activity	Mechanism of action	References
1	Broad‐spectrum antibacterial and antifungal activity	Plumbagin demonstrates significant activity against both Gram‐positive and Gram‐negative bacteria, as well as fungal species such as *C. albicans*	Chen, Yin, Wu, et al. (2020)
		Disruption of bacterial membranes, Induction of reactive oxygen species (ROS) leading to oxidative stress Inhibition of quorum sensing and biofilm formation, which is critical in chronic infections and antibiotic resistance	Razavi et al. (2023)
		Synergistic effects with antibiotics	Chen, Yin, Peng, et al. (2020)
2	Antiviral	Inhibition of viral replication Plumbagin has demonstrated inhibitory effects on replication of viruses such as herpes simplex virus (HSV) and has shown potential activity against HIV by interfering with viral enzymes and disrupting the redox balance necessary for viral survival	Susithra et al. (2016)
		Immunomodulatory effects Plumbagin modulates immune responses by influencing cytokine production and inflammatory signaling pathways, which may enhance host resistance to viral infections	Aronsson et al. (2016)

### 3.3 Antifungal Activity

**FIGURE 4:**
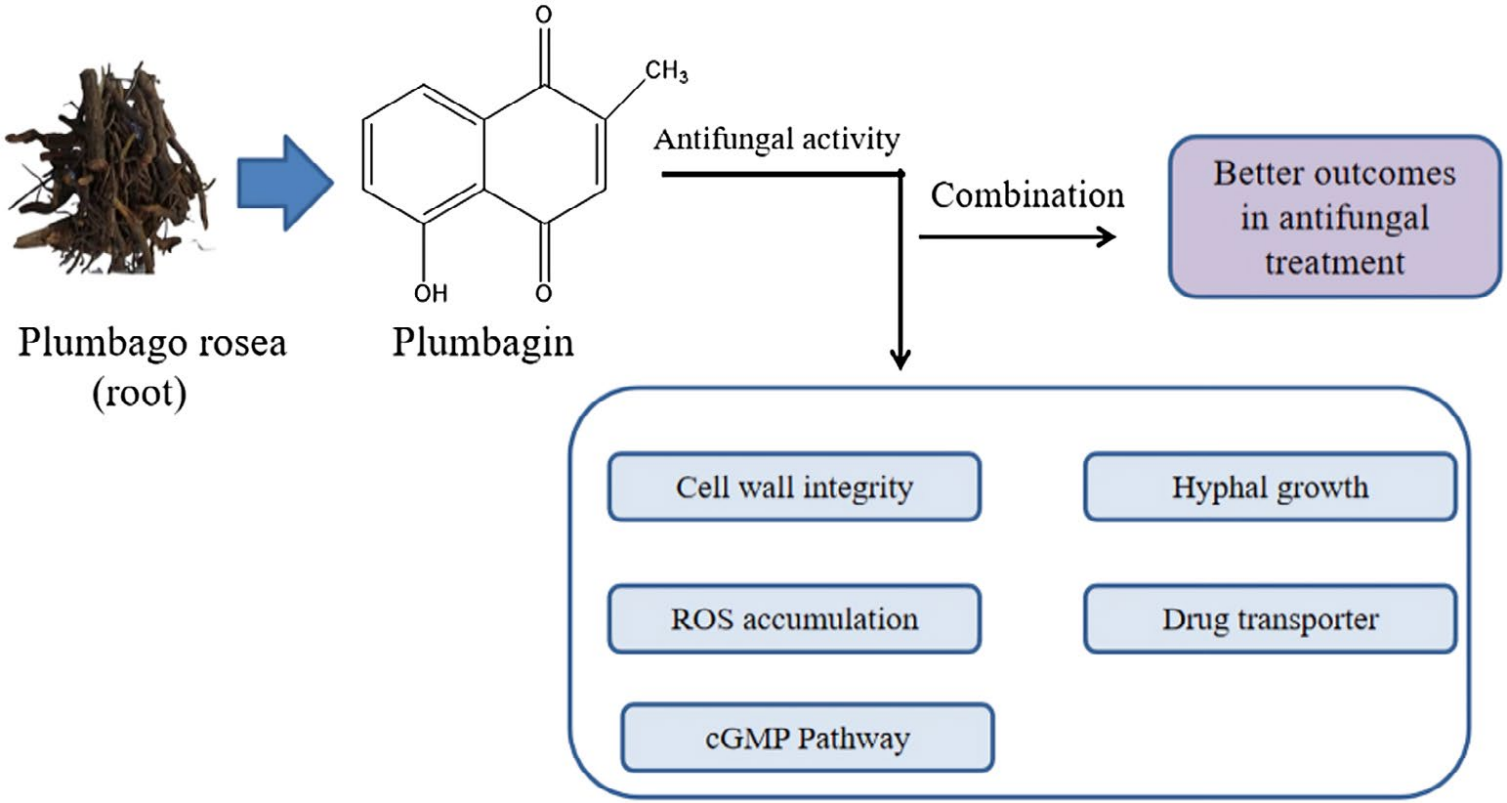
Plumbagin mediated molecular mechanism of antifungal activity.

PB inhibits the growth of 12 filamentous fungi and yeast pathogens, including *Aspergillus flavus*, *A. niger* , *Alternaria* sp., *C. albicans* , *C. glabrata* , and *C. krusei* , as well as *Cryptococcus neoformans* , *C. tropicalis* , *Cladosporium*, *Geotrichum candidum*, *Fusarium*, and *Penicillium*. Comparing PB to the control medication ketoconazole, this study indicated PB to be a potential antifungal agent (Dzoyem et al. 2007). Mahoney et al. (2000) investigated the effects of aflatoxigenesis and fungal vitality of walnuts on four naphthoquinones: juglone, menadione, PB, and 1,4‐naphthoquinone. At higher concentrations, quinones reduced *A. flavus* growth and delayed germination. Aflatoxigenesis and decreased fungal vitality have been associated with structural features including 2‐methyl and 5‐hydroxyl substituents. PB has been shown to inhibit *Pleurotus sajor‐caju*, a white‐rot basidiomycete, from growing on malt agar plates (Curreli et al. 2001) (Figure 4).

### 3.4 Anti‐Inflammatory and Antioxidant Properties

**FIGURE 5:**
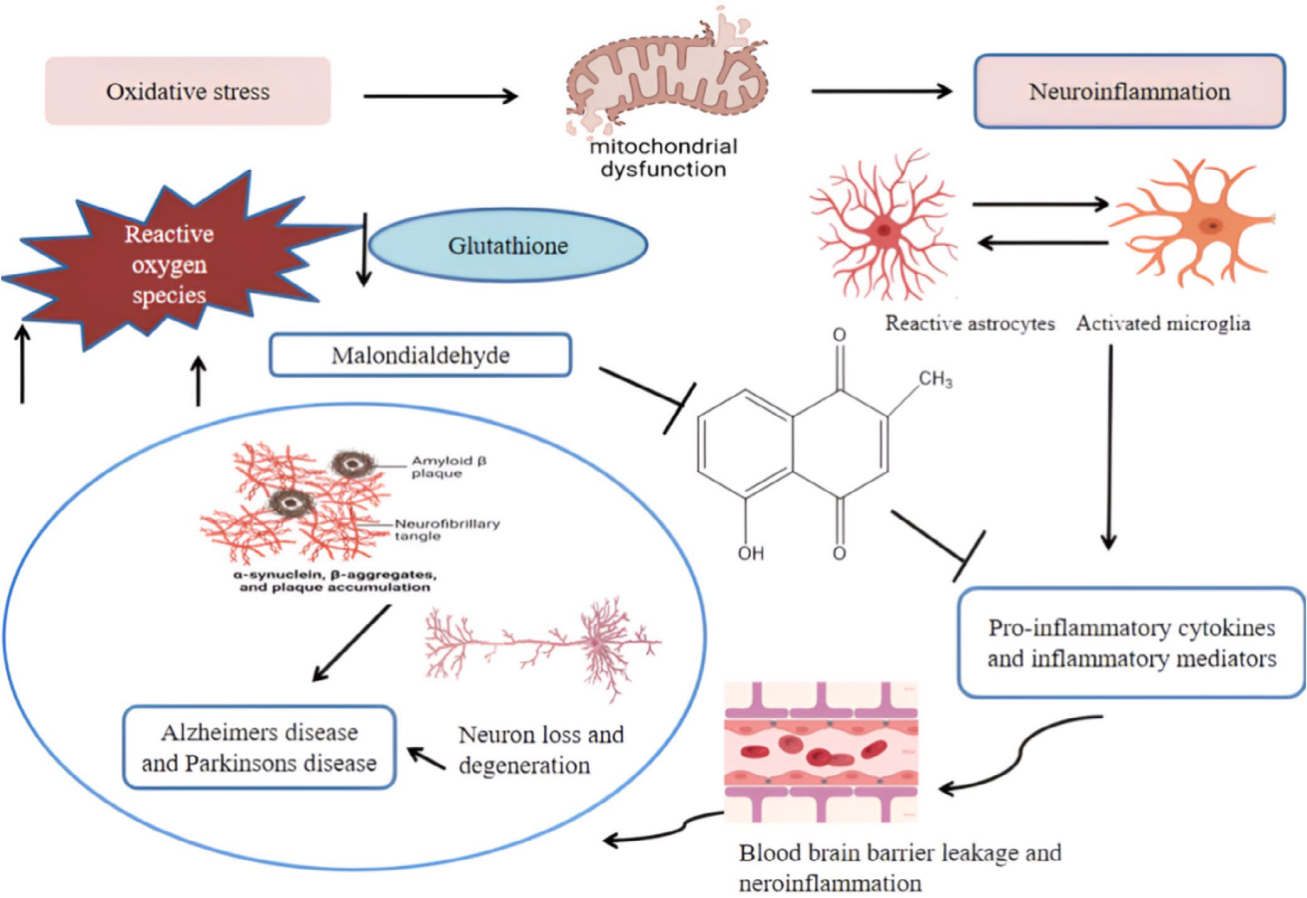
Molecular mechanism of antioxidant and anti‐inflammatory activity by plumbagin.

Plumbagin has numerous pharmacological and therapeutic benefits. The anti‐inflammatory and antioxidant properties of PB help treat a wide range of ailments. PB has been found in various well‐researched in vitro and in vivo pharmacological studies to have positive antioxidant and anti‐inflammatory activities. Unbalanced levels of ROS and antioxidants promote oxidative stress, resulting in mitochondrial failure and increased astrocyte and microglia activation. These cells generate chemokines and inflammatory cytokines that encourage neuronal death and cellular apoptosis (Luo et al. 2024). Numerous pathogenic mechanisms, including oxidative stress, inflammation, imbalanced neurotransmitters, mitochondrial dysfunction, apoptosis, and hereditary factors, can cause neuronal degeneration. Numerous studies have indicated the importance of inflammation is important (Sukkasem et al. 2016). These include oxidative stress and neurodegenerative disease. PB has been shown in animal models of Alzheimer's disease to reduce neuroinflammation and oxidative stress by increasing GSH levels, inhibiting lipid peroxidation and ROS production, and lowering the levels of pro‐inflammatory cytokines (TNF‐α, IL‐6, IL‐1β, and IL‐1α), MDA, iNOS, NF‐κB, COX‐2, and NO. PB‐supported mice with behavioral and neurochemical deficiencies by lowering inflammation (IL‐1β) and oxidative stress (MDA, nitrite) (Poprac et al. 2017). In neurodegenerative disorders, PB may possess antioxidant and anti‐inflammatory properties, and the production of free radicals might result in neuroinflammation and nitro‐oxidative stress. It is thought that the underlying cause of neurobehavioral problems is free radicals, which produce neuroinflammation and nitro‐oxidative stress (Kumar and Pandey 2015) Plumbagin's anti‐inflammatory activity contributes to its potential therapeutic effects in managing chronic inflammatory conditions such as rheumatoid arthritis (RA). Plumbagin inhibits immune cell infiltration, reduces cytokine levels, and decreases joint inflammation in animal models of RA. Inflammatory Bowel Disease (IBD)‐ In experimental models of colitis, plumbagin decreases myeloperoxidase activity, inhibits inflammatory mediators, and enhances colon histology. In neurodegenerative diseases and neuroinflammation, plumbagin lowers the release of inflammatory cytokines and microglial activation, both of which are linked to neurodegenerative illnesses such as Parkinson's and Alzheimer's. Because plumbagin inhibits NF‐κB, reduces pro‐inflammatory mediators, and regulates oxidative stress, it has anti‐inflammatory properties that make it a potential therapeutic candidate for the treatment of chronic inflammatory illnesses. Nevertheless, additional clinical studies and formulation development (such as nanocarriers) are required to fully realize their therapeutic potential because of the possible cytotoxicity and bioavailability problems. Research has shown that PB has anti‐inflammatory and antioxidant properties in animal models of spinal cord injury, depression, cerebral ischemia, neuropathic pain, and chronic autoimmune diseases, including encephalomyelitis. For it to function, inflammatory mediators and ROS are inhibited (Verma and Goyal 2024) (Figure 5).

### 3.5 Anti‐Hypertension Activity

**FIGURE 6:**
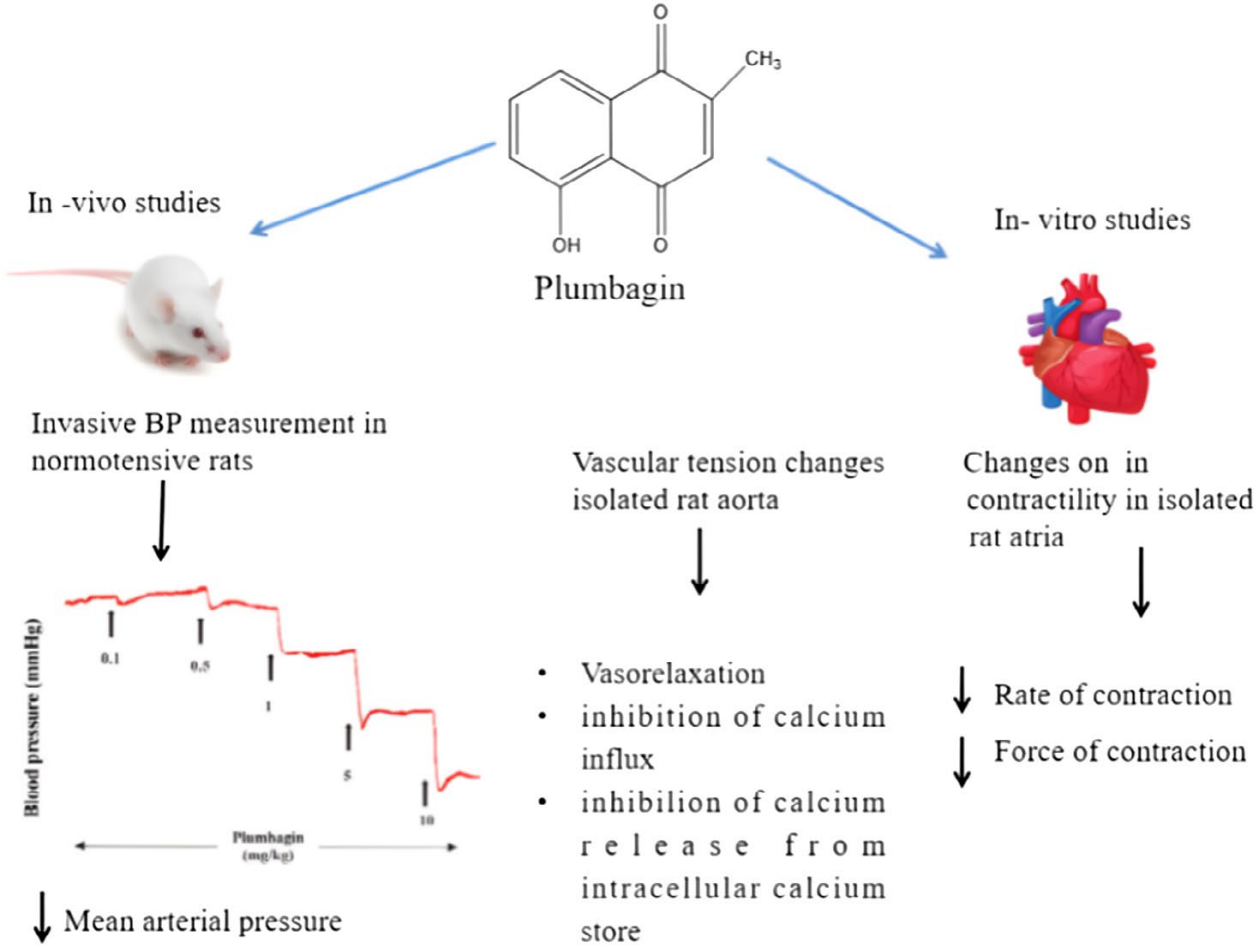
Plumbagin mediated mechanism of antihypertensive activity.

This study used both in vitro and in vivo approaches to investigate the ability of plumbagin to reduce blood pressure in rats. The indicator of high blood pressure is that heart output and vascular resistance increase when vascular smooth muscle cells contract or relax, according to a study by Foëx and Sear (2004). The effectiveness of plumbagin in reducing blood pressure was examined in this study using an invasive blood pressure (IBP) instrument, which is the gold standard for measuring blood pressure (Parasuraman and Raveendran 2012). The isolated rat thoracic aorta was used to evaluate vascular tension changes, whereas isolated right atrial strips were used to determine cardiac depressant effects based on the contraction force and rate. Plumbagin was administered intravenously to normally anesthetized patients, and the dose‐dependent effect of plumbagin was demonstrated by a notable reduction in mean arterial pressure (MAP) in rats administered the injection intravenously while under standard anesthesia. PB reduced the MAP by approximately 34% when administered at a dose of 10 mg/kg. The vascular pathways that PB uses to reduce blood pressure were studied in vitro on aortas that had been isolated from rats. PB was evaluated in restricted aortic rings with intact or injured endothelial cells to ascertain whether it operates through endothelium‐dependent or endothelium‐independent mechanisms. Phenylephrine and high Kþ vasoconstrictors caused plumbagin to respond in a distinct biphasic manner, inducing a brief rise in aortic tissue vasoconstriction followed by progressive relaxation. It has been demonstrated that plumbagin increases the contraction of the renal and mesenteric arteries in rats when phenylephrine is applied (Kim et al. 2017). By binding to α1‐receptors in the smooth muscle, phenylephrine triggers G‐protein‐coupled processes that activate IP3 and DAG. Calcium is subsequently released from intracellular reserves and enters the extracellular calcium channels as a result. High Kþ levels stimulate calcium influx via voltage‐dependent L‐type Ca^2+^ channels, causing vascular smooth muscles to contract (Qamar et al. 2018). According to the findings, PB may act on voltage‐dependent calcium channels (VDCC) to limit calcium influx or restrict calcium release from intracellular reserves. It decreased the contractions caused by elevated Kþ and PE in aortic preparations. Calcium chloride (CaCl_2_) concentration response curves (CRCs) in an EDTA‐Krebs solution with and without 30 μM PB and without Ca^2+^ were made in order to evaluate the compound's efficacy against voltage‐dependent calcium channels (VDCCs). These novel findings about the hypotensive qualities of PB may indicate its possible use in the management of hypertension. Previous studies have demonstrated the antioxidant, anti‐inflammatory, anti‐atherosclerotic, and cardioprotective properties of PB. PB exerts its antihypertensive effects and the mechanisms involved in its potential to regulate blood pressure. Recent research has shown that plumbagin, a naturally occurring naphthoquinone, exerts antihypertensive effects through several mechanisms. Plumbagin prevents calcium from entering vascular smooth muscle cells and causes vasodilation. In vitro tests using rat aortic rings demonstrated that plumbagin shifted the calcium concentration‐response curves to the right, which is comparable to the effect of the well‐known calcium channel blocker nifedipine. Accordingly, plumbagin lowers blood pressure by acting as a calcium antagonist and by lowering vascular resistance. Plumbagin decreased contraction power and heart rate in isolated rat atrial tissues. The fact that pretreatment with atropine or atenolol did not alter these effects suggests that plumbagin acts without the assistance of muscarinic and beta‐adrenergic receptors. One of the ways to lower blood pressure is by decreasing cardiac output. The vasorelaxant effects of plumbagin were apparent even after the vascular endothelium was removed and were not affected by the inhibition of guanylate cyclase (using methylene blue) or nitric oxide synthase (using L‐NAME). This suggests that its vasodilatory activity is independent of the NO pathway originating from the endothelium. Inhibition of Intracellular Calcium Release: Plumbagin clearly inhibited phenylephrine‐induced contractions in rat aortic rings in the absence of calcium, indicating that it obstructs intracellular calcium release, which adds to its vasodilatory effects (Ahmad et al. 2022). Future studies on the effects of PB on hypertension and cardiovascular illnesses may be possible given its features, vasorelaxant action, and adverse chronotropic and inotropic effects (Ahmad et al. 2022) (Figure 6).

### 3.6 Anti‐Ulcer Activity

This study focused on *P. zeylanica* antiulcer; the aqueous root extract of *P. zeylanica* offers similar protection against aspirin‐induced and indomethacin‐induced ulcers, in contrast to the well‐known antiulcer drug omeprazole. *Plumbago zeylanica* may protect against stomach lesions caused by aspirin and indomethacin by inhibiting 5‐lipoxygenase activity (Shaikh et al. 2015). Furthermore, the protective action of the extract may be linked to the phytochemical compounds identified in many reports from previous studies (Shaikh et al. 2015). *Plumbago zeylanica* roots contain astringent tannins, which could account for the antimicrobial effect and support an antiulcer effect. Lipids containing unsaturated fatty acids and many double bonds are particularly susceptible to the effects of free radicals among biological substances. Lipid peroxidation damages cellular membranes and causes significant structural and functional damage. Lipid peroxidation is being intensively investigated in relation to disease and its control by antioxidants in various contexts. This procedure generates numerous by‐products that affect locations other than those where they are produced; consequently, they behave as toxic secondary messengers. Oxidation leads to lipid degradation, often known as rancidity (Li et al. 2025). Fission of cyclic endoperoxidases produces malondialdehyde (MDA). MDA and thiobarbituric acid were combined to make an addict. The selected plants exhibited antioxidant activity, supporting an anti‐free radical mechanism.

Therefore, extracts may serve as antioxidants to prevent the overproduction of reactive oxygen species (Sun et al. 2023). Ulceration weakens antioxidant defenses, resulting in membrane damage and harmful effects. Research has indicated that plants with high antioxidant activity can decrease free radicals in biomolecules. This species, with its antioxidant activity, demonstrated a preventative intervention. The stomach's acidic interior is caused by “stomach acid” (0.1 M hydrochloric acid). Although stomach acid is required for digestion, too much can be uncomfortable. Neutralizing some of the excess acid in the stomach can help relieve stomach acidity; a weak base or “antacid” might be used to neutralize some of it. Acid‐neutralizing capacity (ANC) measures the ability to neutralize acidic inputs. This study attempted to compare the ANC of the extracts. All extracts, particularly *P. auriculata* , exhibited good acid‐neutralizing activity. This study can be compared to other medicinal plants utilized in Ayurvedic therapy. The extract's purported antimicrobial activities could have been valuable in determining its effect on *Helicobacter pylori* , a bacterium that causes peptic ulcers. Tannins have been shown to protect the stomach mucosa by suppressing secretion, which may contribute to the antiulcer properties of the extract. *P. zeylanica* roots also contain flavonoids. They have antioxidant effects and improve mucosal protection by increasing secretion of stomach mucus. Research suggests that boosting mucosal defense has antiulcer properties. This could explain the antiulcer properties of the extracts. Flavonoid extracts have been shown to have antioxidant activity, which is important because oxidative damage is linked to ulcer etiology in both experimental and clinical settings. Antioxidants can prevent lesion formation by ulcer‐related substances. *P. zeylanica* root extract, which is rich in flavonoids and antioxidants, may help to reduce gastrointestinal mucosal damage in animals, as observed in this study.

Ethanol‐induced ulcers are commonly used to evaluate gastroprotective efficacy. Ethanol generates free radicals by promoting the production of leukotrienes and mast cell secretory products. In reality, ulcers are most common in the glandular portion of the stomach. Free radicals play a role in scavenging mucosal ulcers and ultimately in healing them. In the current study, the test group experienced a much lower incidence of ulcers than the control group. The ability of the herbal extracts to prevent ulcers in both models suggests that they have cytoprotective properties. The gastric mucosa's production of prostaglandin may have acted as an antioxidant, scavenging free radicals. The study found that the plant extract has equivalent antiulcer activity to the control. The extract of *P. auriculata* without PB showed antiulcer action. This study assessed oxidative stress, acidity, NSAIDs, and ethanol levels in PB. Thus, this herb may have antiulcer properties. In vitro studies have indicated that *P. zeylanica* and *P. indica* have significant antiulcer properties. PB, which is known for its antioxidant and acid‐neutralizing properties, can effectively treat ulcers.

**FIGURE 7:**
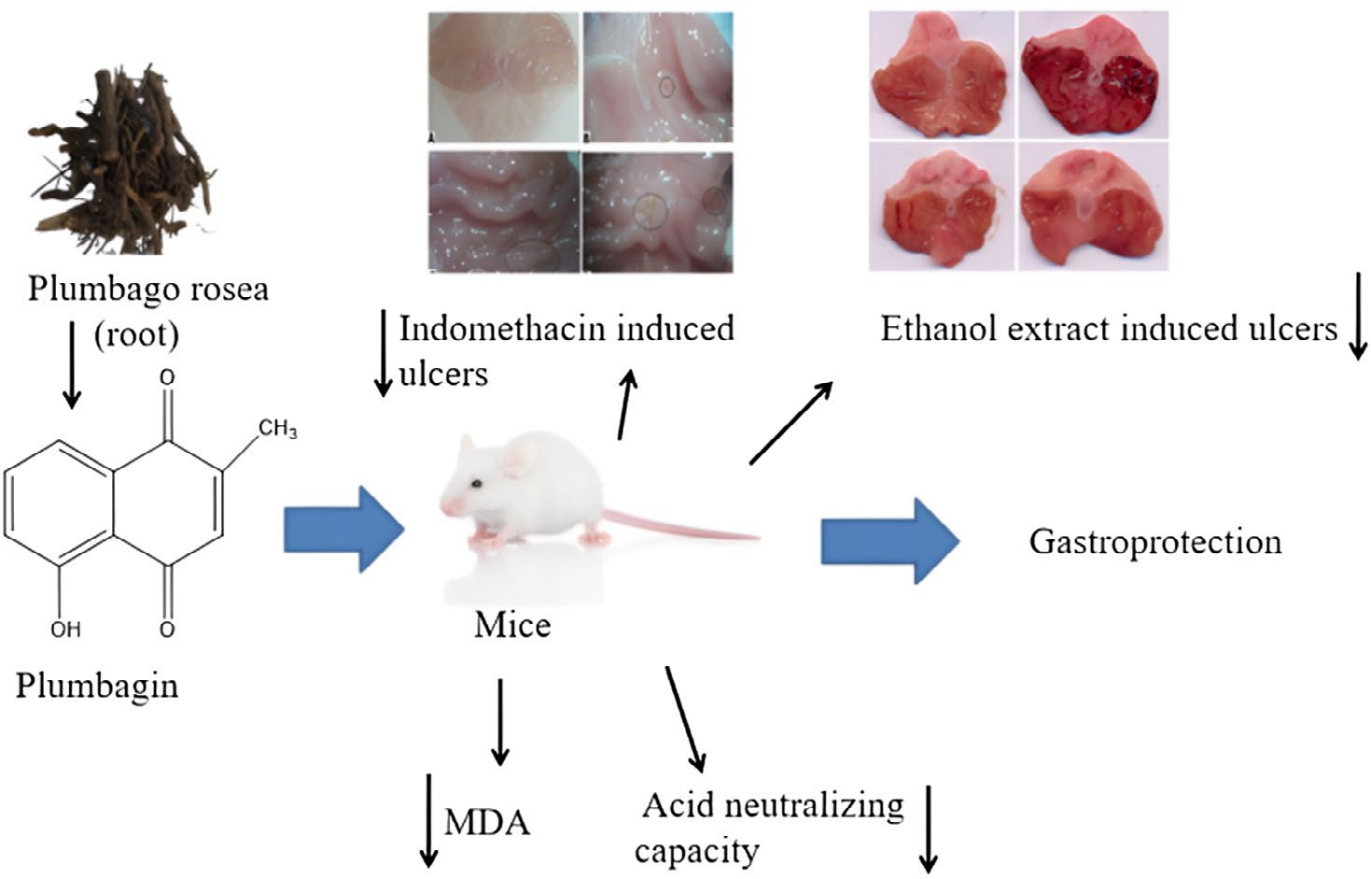
Antiulcerative mechanism of Plumbagin activity.

Phytochemical compounds such as tannins contribute to the antiulcer and antimicrobial effects of *P. zeylanica* . The astringent tannins in *P. zeylanica* roots have the ability to shield the stomach mucosa. By covering the mucosal lining with a protective layer, irritation is reduced and healing is encouraged. Tannins have also been shown to reduce stomach secretions, which may help to prevent and treat ulcers. Flavonoids are another class of phytochemicals with antioxidant properties that contribute to the antiulcer effect by promoting the secretion of stomach mucus and strengthening the mucosal defense system (Han et al. 2025). Tannins, flavonoids, alkaloids, and saponins are among several phytochemicals that give *P. zeylanica* its antibacterial properties against *P. zeylanica* . These substances can break down microbial cell membranes and prevent certain infections from growing. Extracts from various plant parts, such as the roots and leaves, have been shown to have strong antibacterial activity against a variety of bacteria, including *Bacillus subtilis* and *Staphylococcus aureus*. Methanol and ethanol extracts exhibit significant outcomes, but their effectiveness varies depending on the extraction solvent used (Ahmad et al. 2022). *Plumbago* species are being studied for their gastro‐protective benefits, particularly antiulcer activity (Falang et al. 2012) (Figure 7).

### 3.7 Cognitive Function and Neuroprotection

**FIGURE 8:**
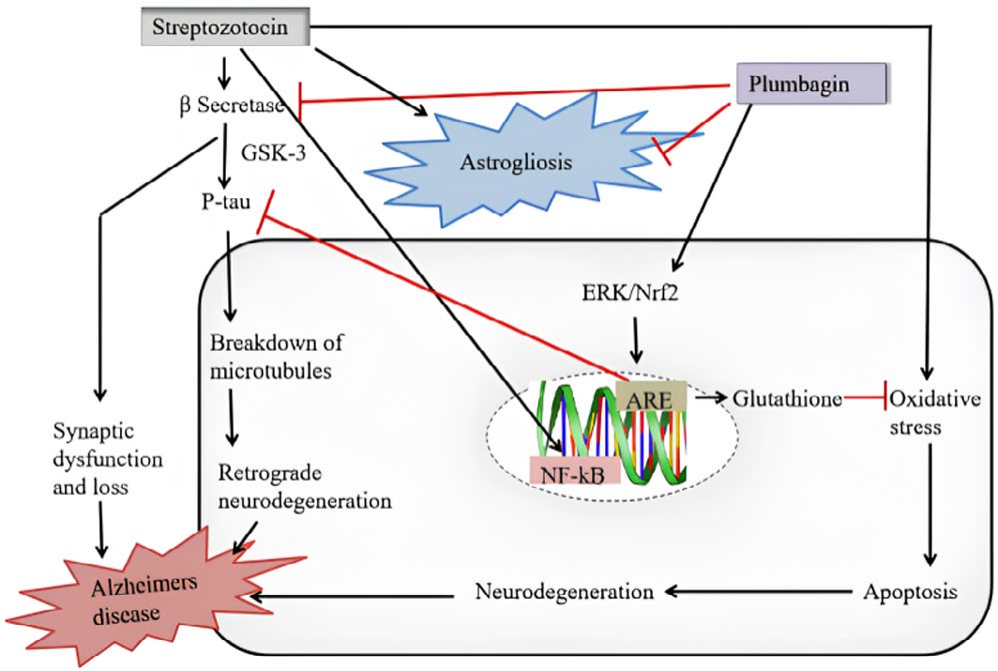
Molecular mechanistic neuro‐protection activity by Plumbagin.

According to recent studies, PB may have neuroprotective properties. By activating the Nrf2/ARE pathway, it prevents brain ischemia, oxidative stress from spinal cord damage, and inflammation. By altering brain‐derived neurotrophic factor, plumbagin prevented isoflurane‐induced hippocampal neuronal loss. Signaling pathways (Son et al. 2010; Zhang et al. 2015) including BDNF–TrkB–PI3/Akt and ERK/JNK (Yuan et al. 2017) have been implicated in neuroprotection. PB boosted neuroprotective chemicals such as glutathione in stressed mouse brains (Trepanier and Milgram 2010). Neuroinflammation causes neurodegeneration, leading to disorders such as Alzheimer's disease (AD). PB effectively reduces neuroinflammation in Alzheimer's disease by inhibiting COX‐2 (Trepanier and Milgram 2010). PB inhibits NF‐κB activity, which lowers inflammation. In AD (Lukiw 2012), pro‐inflammatory cytokine genes are activated by NF‐κB, leading to neuroinflammation. In BV‐2 microglial cells stimulated by lipopolysaccharide, PB reduces pro‐inflammatory cytokines. PB has recently been utilized in the treatment of neurological illnesses, such as Huntington's, Parkinson's, and Alzheimer's disease. PB showed neuroprotective effects and improved memory performance. Researchers have studied the potential to improve AD symptoms. This chronic neurological condition causes progressive deterioration of cognitive capacity, such as memory and thinking. In the Morris water maze, PB significantly improved memory and learning in mice with STZ‐induced AD. Trigonelline, an inhibitor of the Nrf2/ARE pathway, lessened PB effects (Figure 8).

## 4 Plumbagin Derivatives

**FIGURE 9:**
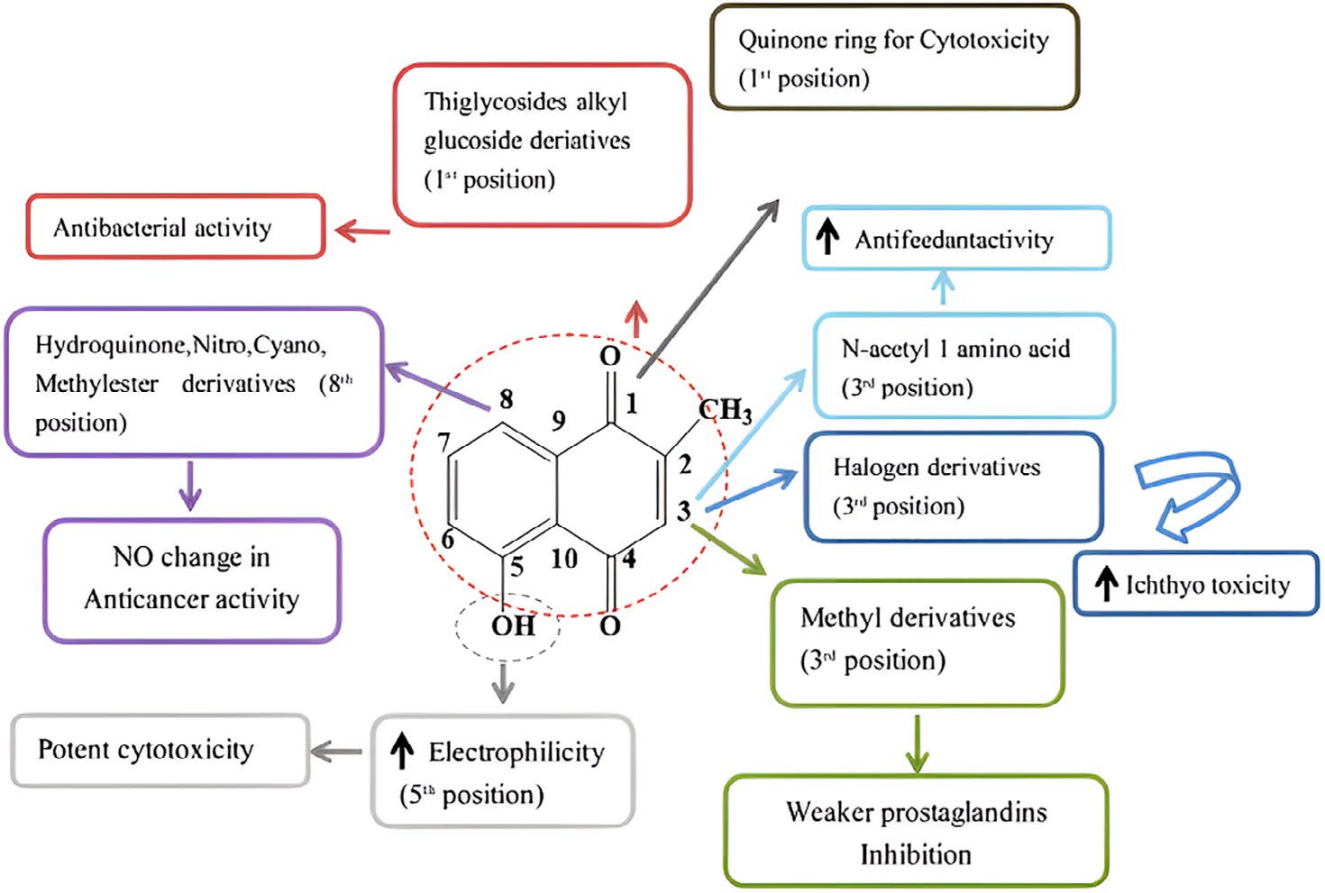
Structural changes of plumbagin derivatives and their pharmaceutical activities.

They were created to enhance their pharmacokinetic properties and bioavailability (Figure 9). The N‐acetyl‐l‐amino acid side chain was substituted at position three, considerably increasing the antifeedant's ( *Spodoptera litura* ) activity. PB inhibits human DNA topoisomerase II. When a molecule has a quninone ring, electron transport may produce radicals that can be harmful. One possible explanation for the cytotoxicity of PB is its unusual structure, which includes a quinone ring. Tumor‐inhibitory action was not significantly improved by PLB's hydroquinone, nitro, cyano, or methyl ester derivatives. The reduction of naphthoquinone and amide derivatives from 1,4‐naphthoquinone carboxylic acid in PB inhibited trypanothione reductase (TR) from *Trypanosoma cruzi*, indicating trypanocidal action. The 3‐methyl derivatives of PB homologues (2‐alkyl‐1,4‐naphthoquinones) exhibited less potent inhibition of prostaglandin synthetase (PGS). The third‐position halogen substitution enhanced ichthyotoxicity (*Poeculia reticula*) in contrast to free PB. Thioglycosides and derivatives of alkylglucosides inhibit two enzymes involved in mycothiol synthesis in bacteria: N‐acetylglucosaminylinositol (GlcNAc‐Ins)‐deacetylase (MshB) and mycothiol‐S‐conjugate amidase. Phenyl‐2‐deoxy‐2‐amino‐1‐thio‐a‐D‐glucopyranoside, a potent MshB inhibitor, is formed via the formation of an amide bond and two to five methylene carbons at the C‐3 position. The inhibitory action of MshB on PB was demonstrated by significantly changing the carbon chain length. PB's hydroxyl group enables it to absorb electrons and generate reactive oxygen species, which increases its electrophilic properties and cytotoxicity (Rajalakshmi et al. 2018).

### 4.1 Bioavailability of Plumbagin

Plumbagin is a biological agent with high activity, and investigations have been conducted to determine its bioavailability. Hsieh et al. (2006) employed liquid chromatography–tandem mass spectrometry (LC–MS/MS) and automated blood sampling to determine the oral bioavailability of PB in rats. Following separation on a C18 column (150 mm × 4.6 mm I.D.; 5 mm), the rat plasma was eluted with a mobile phase consisting of water‐acetonitrile (40:60, v/v). PB analysis utilized multiple reaction monitoring to track the transition from the deprotonated molecule *m/z* 187 [MH](~) to the resulting ion *m/z* 159 [MHCO](~). Eighty (80%) recovered PB was detected in rat plasma, with detection values of 5 ng/mL and quantification limits of 10 ng/mL. According to pharmacokinetic studies, the oral bioavailability of PB in rats is 38.775%. The researchers employed LC–MS/MS to determine the excretion of PB and its metabolites in the urine and feces of rats administered oral therapy. In mice administered an oral dose of 100 mg/kg, 49% of the drug was removed through the feces. PB maximum serum concentration (*C*_max_) was 0.35 mg/mL an hour later, but it quickly dropped after that. An AUC of 271.9 mg/kg was determined, with a *T*_max_ (min) of 150. PB metabolites with molecular weights of 202, 362, and 536 kDa were found in rat urine. These findings indicated that the PB metabolic pathway may include phase I aliphatic hydroxylation (MW 202) and phase II glucuronidation (MW 362, 536). Mandala Rayabandla et al. (2010) evaluated the antitumor efficacy and systemic toxicity of free PB in chitosan‐based microspheres. Pharmacokinetic studies showed that the PB elimination half‐life increased by 22 times, reducing tumor development and systemic toxicity. The authors proposed chitosan‐based plumbagin microspheres for systemic administration. Plumbagin can be found in the urine of rats four hours after injection, although it may not be present in blood for up to twenty‐four hours. Some drug residues persisted in the urine for up to 48 h, but the bulk was removed after 24 h. The authors suggested that the anticancer actions of this molecule may be due to a reaction initiated by the parent chemicals (Rajalakshmi et al. 2018).

### 4.2 Drug Delivery of Plumbagin

According to animal studies, PB has limited biopharmaceutical characteristics such as high lipophilicity, low melting point, short biological half‐life, and insolubility in water, leading to only 39% oral bioavailability (Bothiraja et al. 2013). It is regarded as a very dangerous quinine moiety that functions as a spindle poison, exhibiting cytotoxic and radiomimetic effects at higher concentrations, and preventing cell mitosis at lower concentrations. Because of its competitive inhibition of vitamin K action, PLB administration over an extended period may cause bleeding. To increase the therapeutic efficacy in light of these limitations, researchers have developed a range of drug delivery strategies.

#### 4.2.1 Liposomes

Liposomes are natural phospholipids such as cholesterol, which are formed into small spherical vesicles. They improve pharmacokinetics, biodistribution patterns of active moieties, intracellular absorption of components, solubility, bioavailability, and stability in vitro and in vivo (Bardania et al. 2017). Liposomes are preferred by the pharmaceutical and cosmetic sectors because of their unique properties, which include low toxicity, site specificity, biocompatibility, biodegradability, and the ability to entrap both hydrophobic and hydrophilic substances (Panahi et al. 2017). Documentation in the literature indicates that a number of liposomal products are available, many of which are undergoing various stages of clinical testing. In addition to the previously mentioned factors, liposomal delivery techniques have been developed to resolve the problems related to PB's therapeutic potential. These factors included flexibility and compatibility. Tiwari et al. (2002) used a thin film hydration technique to create thermosensitive tiny unilamellar liposomes filled with DPPC and DSPC. At 42°C, liposomes were released, and at 37°C, less than 5% were released. Drug entrapment was made possible by the liquid crystalline fluid state of the liposome bilayer at high temperatures, and when used in conjunction with treatment for localized hyperthermia, the liposomes also enhanced the tumor response in mice (Tiwari et al. 2002). Sunil Kumar et al. (2011) created PLB‐loaded extended circulating pegylated liposomes to improve plasma half‐life and anticancer potential utilizing B16F1 melanoma as a solid tumor model. The formulation exhibited improved zeta potential, cumulative drug release, efficiency, and particle size. It also reduced tumor volume significantly in B16F1 melanoma cells growing in vivo. By forming a hydrophilic covering, the pegylation process prevented opsonin from penetrating and adhering (Sunil Kumar et al. 2011).

#### 4.2.2 Niosomes

Niosomes are vesicles composed of nonionic surfactants that form closed bilayer structures when they self‐assemble in an aqueous environment. Nonionic surfactants, such as polyoxyethylene alkyl ethers, sorbitan monoesters (Span 20, 40, 60, and 80), and polyoxyethylene sorbitan monoesters (Tween 20, 60, 61, and 80), have been used to create niosomes (Behnam et al. 2018). The niosomal bilayer becomes more rigid and structured when cholesterol is added to the nonionic surfactant. Niosomes are more stable than liposomes and have many benefits over liposomes, such as better patient compliance and therapeutic efficacy when compared to traditional oily formulations. They can also entrap hydrophilic, lipophilic, and amphiphilic medications. Moreover, they can deliver specialized medications to particular organs and shield pharmaceuticals from biological enzymes (Behnam et al. 2018). Oommen et al. (1999) used cholesterol, Span 60, and dicetyl phosphate to hydrate the lipid layer and load PLB‐β‐cyclodextrin (β‐CyD) into niosomes (D'Souza et al. 1998). With a diameter of 12 mm and an entrapment efficiency of 74%, the niosomes had an oval or spherical shape. Diffusion and lipid bilayer erosion caused the hydrophilic PLB‐β‐CyD complex to be released from the niosomes more quickly. When administered subcutaneously to C57BL/6J mice with melanoma B16F1, the niosomal PLB‐β‐CyD complex demonstrated stronger antitumor activity than free PB. Naresh et al. (1996) generated PLB‐loaded niosomes by wetting their lipid layer. The antitumor activity of niosomal PLB was higher in Ehrlich ascites and a solid tumor (sarcoma‐180). LD_50_ survival tests and tumor volume doubling time revealed that niosomal PB was less toxic than free PB and had greater antitumor efficacy in a solid tumor (sarcoma‐180) and Ehrlich ascites model. To boost efficiency and reduce PB toxicity, Kini et al. (1997) developed albumin microspheres and regulated release PLB‐loaded niosomes. Compared to niosomes, albumin microspheres showed exceptional antitumor effectiveness against B16F1 melanoma produced in vivo at a dose of 5 mg/kg, as well as an antifertility effect. Additionally, data on animal survival indicated a slight increase in the survival rate and, consequently, antitumor efficacy. Additionally, histopathological investigations demonstrated that the antiovulatory action changed the antifertility activity.

#### 4.2.3 Polymeric Nanoparticle

Polymeric nanoparticles (NPs) are colloidal solid particles composed of biodegradable polymers and copolymers. Collagen and chitosan are two commonly used polymers, as are non‐biodegradable polymers, such as poly(lactic acid) (PLA) and poly(lactic co‐glycolic acid) (PLGA) (Sah et al. 2018). Multifunctionalized polymeric materials produce dendrimers, micelles, nanocapsules, nanospheres, core shells, polymer matrices incorporating nanoparticles, and other structures. Because they can penetrate capillaries and be absorbed by cells, small particles (50–300 nm) can boost the medication concentration at the site of action (Afsharzadeh et al. 2018). Indhumathi et al. (2013) created PLB‐loaded polymethylcyanoacrylate nanoparticles via emulsion polymerization in a continuous aqueous phase. Polymers such as Span 80, Tween 80, and PEG 4000 were added to the surface of the nanoparticles. The particle size was less than 100 nm, while the entrapment efficiency was 71.63%, indicating that continuous aqueous‐phase emulsion polymerization is the most effective method for producing nanoparticles.

#### 4.2.4 Polymeric Micelle

Bothiraja et al. (2013) used a self‐assembly technique to synthesize phospholipid and Tween 80 mixed micelles, which showed improved anticancer activity. These were investigated as viable injectable nanocarriers for PB. The combined micelles measured 46 nm and were spherical. Zeta potential was 5.04 mV, drug loading was 91.21, and encapsulation efficiency was 98.38%. PLB's in vitro antitumor activity against human breast cancer MCF‐7 cells was 2.1 times higher in micelles with sustained release than in the free form. PB was safe for intravenous administration because it remained stable at high pH, retaining micelle size and encapsulation effectiveness after dilution. Loading PB into D‐α‐tocopheryl polyethylene glycol 1000 succinate (vitamin E TPGS1k or TPGS)‐folic acid conjugate nanomicelles (also known as PTFM) reduces toxicity and allows for targeted distribution with synergistic anticancer efficacy. Loading PB into TPGS micelles without folic acid conjugates (also known as PTM) or PTFM reduced size and improved encapsulation efficiency. PTM and PTFM had longer circulation times, slower plasma clearance, no evidence of tissue or blood toxicity, and increased PB bioavailability by 3.8 and 4.8 times, respectively, when compared to free PB. In vitro, micelles had a greater anticancer effect on human breast cancer MCF‐7 cells with high folate levels. PTFM has a targeting effect. The medication concentration (GI50) needed to block 50% of cell growth at a specific time period (PB) is 13.15 ± 1.31 μg/mL, while it reduces by 40.68% with PTM. Additionally, PTFM has a GI50 value of 3.2 ± 0.4 μg/mL, which is 75.67% lower than free PB. TPGS and PB interact synergistically. The polymeric micellar drug delivery system is a revolutionary approach that reduces dosage and costs by utilizing a carrier with therapeutic properties (Pawar et al. 2016).

#### 4.2.5 Cyclodextrin Complexes

A lipophilic core and hydrophilic outer shell characterize cyclic oligosaccharides, known as cyclodextrins (CDs). The three most common CD types are α‐, β‐, and γ‐CD. The solubility, bioavailability, tolerance, and elimination of unwanted side effects of pharmaceuticals are all improved by the bioadaptability and multifunctional qualities of CDs. Numerous lipophilic medications can be hosted by CD complexes (Gorjikhah et al. 2017). CD complexes, which also offer a variety of platforms for cutting‐edge drug delivery techniques. Using the neutralization approach, Singh and Udupa (1997) produced a 1:1 M ratio inclusion combination of PB and betacyclodextrin. The complex and plain PLBs had respective LD50 values of 12.88 and 8.51 mg/kg body weight, respectively. The combination increased the antitumor effectiveness of PB while decreasing its toxicity, and the enhanced antitumor efficacy of complexation improved PB solubility at the same dosages as pure PB, enabling it to be absorbed more quickly in the bloodstream and have a greater antitumor effect.

#### 4.2.6 Metal Nanoparticles

Solid metal particles containing medicinal ingredients are known as metallic nanoparticles. They can be attached to a surface, enclosed in a shell, or dispersed in a polymer carrier matrix. They have become cutting‐edge tracking and contrast agents in many cancer treatments. The stability and bioavailability of medicinal substances are enhanced by the high surface area‐to‐volume ratios of metallic nanoparticles, which also make chemical modification easier and allow drugs to enter cellular compartments. They also protect pharmaceuticals from harmful biological environments. Plumbagin has been encapsulated in metallic nanoparticles. A pH‐responsive bone‐targeting drug delivery system is required for precise and sensitive diagnosis and treatment of bone metastases. MNP‐PL, or plumbagin‐loaded magnetic nanoparticles, have been developed in a novel manner, and plumbagin‐loaded magnetic nanoparticles (MNP‐PL) (Shahida Parveen et al. 2014) have been developed and tested in a novel pancreatic cancer treatment formulation.

Although the toxicity and bioavailability of the MNP‐PL formulation were low, it offered a four‐fold dose advantage over regular plumbagin. The mechanical, thermal, and physical characteristics of collagen scaffolds were enhanced by caged plumbagin on silver nanoparticles (PCSN). By altering the redox signaling pathways of cancer cells, PB‐AgNPs (Hafeez et al. 2016; Duraipandy et al. 2014) were engineered to specifically kill them. In order to reduce plumbagin's toxicity, cellular localization, and reactive oxygen species production, plumbagin‐conjugated gold nanoparticles (AuPB) were created (Bothiraja et al. 2013).

#### 4.2.7 Conjugation With Polymers

Newton et al. (2010) reported that the 5‐hydroxy functionality was directly electro‐oxidized to produce a PB film on a carbon substrate. This film is employed as an entrapment matrix in biosensing applications. The film was anticipated to generate a polyphenylene oxide film that was primarily nonconductive, in line with standard phenolic electrooxidation. With the development of a phenol layer, cyclic voltammograms demonstrated that the phenol underwent irreversible oxidation at +0.95 V and declined with subsequent scans. One effect of scan number on the quinone component profile was the movement of the quinone reduction peak to higher negative potentials. The electrode surface retained the quinone component's activity. A gold‐patterned crystal was used to repeat the polymerization process in order to boost the monomer concentration and facilitate the deposition of a thicker, maybe more active coating. The stability of the film was evaluated by reacting it with typical biological agents such as glutathione.

Serum albumin and medication interactions are essential for creating novel treatments (Chrastina et al. 2020). Because of its high reactivity with cysteine and nucleophiles containing cysteine, the spontaneous PB‐albumin adduct is expected to form a covalent connection between carbon C3 of PB and cysteine 34 of albumin. PB instability, high lipophilicity, and low solubility are resolved by this binding, which may restrict its therapeutic application. Conjugation of PB with albumin may improve its pharmacokinetic profile, eliminating the need for new formulations and delivery technologies, such as liposomes, niosomes, microspheres, nanoparticles, micelles, complexation, metal nanoparticles, and crystal modification.

In addition to Dandawate et al. (2014), this study synthesized and characterized a novel cyclodextrin inclusion complex of plumbagin‐isoniazid (PLIHZCD), which upon cyclodextrin complexation exhibits enhanced water solubility and potent antitubercular action. Under low‐iron conditions, which correspond to the isoniazid‐resistance scenario, the compound shows better action than isoniazid. According to the phase solubility investigation, PLIHZ's aqueous solubility increases linearly with cyclodextrin content. PLIHZ was discovered to have an aqueous solubility of 32.23 µg/mL, which significantly increased to 60.97 µg/mL upon complexation with β‐cyclodextrin.

#### 4.2.8 Problems With Bioavailability

Plumbagin has low water solubility and high lipophilicity, which results in restricted systemic bioavailability and poor gastrointestinal absorption. Its decreased effectiveness in animal models in comparison with in vitro results may be due to the fact that oral treatment only yields a bioavailability of roughly 39%, according to in vivo studies (Sumsakul et al. 2014). Plumbagin has a short elimination half‐life of roughly five hours and delayed absorption according to pharmacokinetic studies conducted in rats (median time to peak plasma concentration of 9.63 h). These elements restrict the therapeutic window. Dose‐dependent toxicit—acute toxicity studies in rats have shown that a single oral dose of 150 mg/kg is the most tolerated dose; larger doses cause side effects such as agitation, salivation, and anxiety. In subacute experiments conducted over a 28‐day period, 25 mg/kg/day was found to be safe; higher doses caused toxicity and death. Hepatotoxicity, cardiotoxicity, and possible effects on blood coagulation processes have been documented in other studies, indicating that plumbagin toxicity may differ among species and organ systems (Sumsakul et al. 2014).

#### 4.2.9 Methods for Improving Therapeutic Potential

In animal models, it has been demonstrated that protecting plumbagin in niosomes or nanoparticles improves anticancer effectiveness and decreases toxicity. These formulations lessen side effects and improve drug delivery. The production of molecules with enhanced stability and antiproliferative activity against specific cancer cell lines by the synthesis of plumbagin derivatives, such as nitric oxide‐releasing hybrids, suggests exciting potential for therapeutic development (Bao et al. 2017).

### 4.3 Toxicity Study of Plumbagin

PB plant extracts may be a chemopreventive agent that has long been utilized in traditional medicine. Nevertheless, allegations of vesicant and abortifacient qualities have sparked concerns about safety Shukla et al. (2021). In their work, NCI's Chemical Selectivity Group sufficiently addressed these problems, and the literature contained no accounts of PB exposure at work during processing or manufacturing (Singh and Udupa 1997). No studies or case reports have linked human cancer risk to PB exposure. However, adverse side effects, such as diarrhea, skin rashes, and elevated white blood cell counts, have been reported. White blood cell and neutrophil counts, serum and acid phosphatase levels, and liver damage were increased (Singh and Udupa 1997). PB mutagenicity, chromosomal abnormalities, and DNA damage have been investigated using standard methodologies. Farr et al. (1985) found that PB was highly mutagenic in exponential‐phase cells but not in stationary‐phase cells. *E. coli* AQ634 cells were used to determine the frequency of Trp‐ø Trp1 reversal. Santhakumari et al. (1980) investigated the effects of PB on fibroblasts in chick embryos. The number of metaphase cells increased as a result of slower cell development and proliferation and a lower mitotic index. The authors discovered that PB inhibited cells from undergoing mitosis by acting as a spindle toxin at lower concentrations. Nevertheless, it also exhibits radiomimetic cytotoxic and nucleotoxic effects at higher concentrations, and the metabolism, distribution, and excretion of PB have long remained unclear. Chandrasekaran and Nagarajan (1981) introduced a colorimetric technique to assess PB metabolism in rats. According to a recent study (Hsieh et al. 2006), the oral bioavailability of plumbagin in conscious, free‐moving rats was 38.775%, as determined using liquid chromatography‐tandem mass spectroscopy (LC–MS/MS). PB demonstrated substantial antimutagenic efficacy against 1‐nitropyrene (1‐NP), 3‐nitrofluoranthene (3‐NFA), and 2‐nitrofluorene (2NF) in *Salmonella typhimurium* TA (Edenharder and Tang 1997). Evidence suggests that this chemical targets cancer cells precisely and has minimal to no effect on healthy cells. PB effectively inhibits the growth of MDA‐MB‐231 and MCF‐7 BRCA cells and induces apoptosis, while it has no effect on MCF‐10A cells, which are nontumorigenic “normal” breast epithelial cells (Ahmad et al. 2008).

Aziz et al. (2008) found that PB promoted apoptosis in prostate cancer cells (DU145, CWR22rv1, and LNCaP), but had no effect on nontumorigenic immortalized prostate epithelial RWPE‐1 cells. Despite these intriguing results, further preclinical animal studies are needed to improve the ability of PBs to fight cancer. Singh and Udupa (1997) created an injectable poly(lactic‐co‐glycolic) acid (PLGA) gel to improve the anticancer properties of PB while minimizing its toxicity. Through subcutaneous injection, the toxicity of a plumbagin‐containing gel was found to be lower in BALB/c mice than in native PB.

The efficacy of gel implants was demonstrated by their significantly higher volume doubling time (VDT). To reduce toxicity and increase the therapeutic efficiency of plumbagin, researchers have suggested an efficient drug delivery method (Singh et al. 1997). The anti‐infertility and anticancer activities of plumbagin have been studied in controlled‐release formulations using different carriers to increase efficacy and decrease toxicity. Albumin microspheres and niosomes were used as carriers. Unlike niosome microspheres, albumin microspheres at an intraperitoneal dose of 5 mg/kg showed potential anticancer and antifertility effects.

Antitumoral activity and survival rate both somewhat improved, according to animal survival results. The results reported by Naresh et al. (1996) were marginally different. Hydrating the lipid layer allowed the researchers to manufacture plumbagin niosomes. Acute toxicity tests were performed when both free and niosomal PB were administered. Niosomal PB exhibits anticancer properties in Ehrlich ascites and sarcomas. Liposomal PB was found to be a more effective anticancer treatment in C57BL/6J mice with melanoma when paired with localized hyperthermia (431C for 30 min or 1 h) than animals given a similar dose of free PB, with or without hyperthermia. The study drew conclusions by investigating tumor development and VDT. A PB inclusion complex was produced using a neutralization approach and β‐cyclodextrin. More anticancer activity and reduced toxicity were demonstrated by this complex. All naphthoquinones exhibited antimutagenic activity. There was no change in antimutagenic potency when methyl or hydroxyl functional groups were added. Plumbagin performed better than any other drug in the antimutagenicity evaluation (Edenharder and Tang 1997).

**FIGURE 10:**
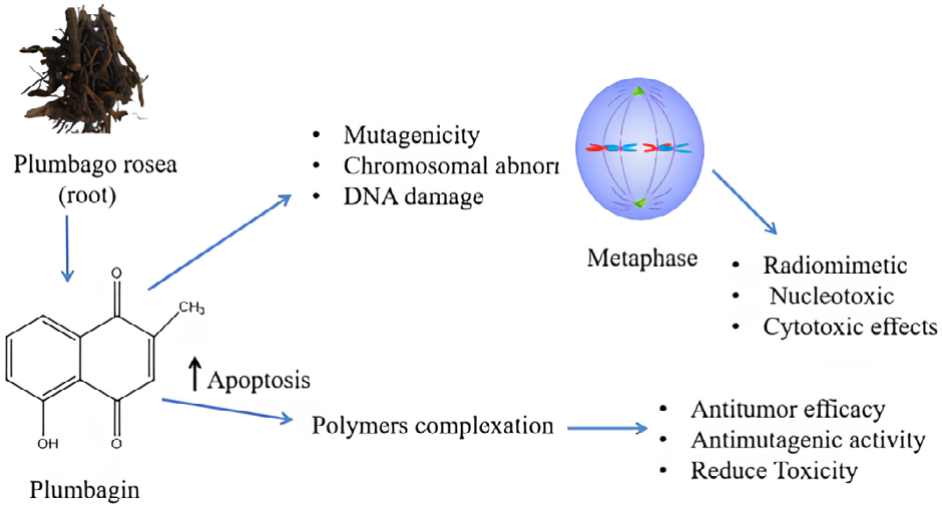
Toxicity profile of plumbagin.

Inbaraj and Chignell (2004) studied the cytotoxicity of plumbagin and its demethylated analog, juglone, against HaCaT keratinocytes. Cell viability was considerably reduced upon exposure to juglone or PB (at concentrations ranging from 1 to 20 mM). Redox cycling and the glutathione (GSH) response are two ways in which quinones affect biology. GSH is oxidized to GSSG by the H_2_O_2_ generated when keratinocytes are incubated with quinones. The increased formation of semiquinone radicals, H_2_O_2_ generation, and cytotoxicity following GSH depletion illustrate the protective effect of buthionine sulfoximine. Both quinones reduced GSH levels in the cells. The stoichiometric conversion of GSH to GSSG by PB suggests that the primary metabolic mechanism is redox cycling. In particular, at higher concentrations (10 and 20 mM), juglone therapy dramatically decreased GSH, which did not appear as GSSG, suggesting that the cytotoxicity of quinone may be due to nucleophilic addition to GSH. This highlights the need for caution when applying topical treatments, including juglone and PB, because they may cause skin damage. Plumbagin, a bioactive naphthoquinone derived from *P. zeylanica*, has anti‐inflammatory, anticancer, and antibacterial properties, making it a promising therapeutic candidate. However, toxicity, bioavailability, and extraction efficiency restrict its clinical use. To increase its therapeutic utility, recent developments have focused on improving extraction techniques and creating plans to reduce toxicity (Tripathi et al. 2025) (Figure 10). The differences in bioavailability and toxicity among various plumbagin analogs and their derivatives are shown in Table 5.

**TABLE 5:** The differences in bioavailability and toxicity among various plumbagin analogues and their derivatives.

Derivative type	Bio‐availability impact	Toxicity impact	Therapeutic potential	References
Esterified analogues	↑ (moderate)	↓ (better selectivity)	Cancer	Ahmed et al. (2014)
NO‐hybrids	↑ (via NO‐release)	↓ (targeted effect)	Cancer, anti‐inflammatory	Bao et al. (2017)
Niosomal/nanoparticles	↑↑ (major increase)	↓↓ (lower systemic)	Cancer	Naresh et al. (1996)
Hydroquinone analogues	↔ (no significant change)	↔ or ↓ (variable)	Less consistent	Hazra et al. (2002)
Drug conjugates	↑ (targeted delivery)	↓ (less résistance)	Antitubercular	Nayak et al. (2014)

### 4.4 Strategies to Reduce Plumbagin's Toxicity

#### 4.4.1 Encapsulation of Niosomes

Niosomes, which are vesicles based on non‐ionic surfactants, have been demonstrated to decrease plumbagin toxicity while increasing its anticancer effectiveness. By increasing drug stability and bio‐availability, this delivery method provides a safer therapeutic profile. Traditional Ayurvedic methods for detoxification (Shodhana) include utilizing lime water to purify *P. zeylanica* roots in order to lower the amount of plumbagin. By successfully reducing toxicity, this procedure makes the plant safer for medical use without sacrificing its therapeutic qualities. The cytotoxic effects of plumbagin have been discovered to be reduced when it is esterified with substances such as lipoic acid. While maintaining or possibly increasing the medicinal efficacy of the compound, these changes also improve its safety profile. While effectiveness studies examine an intervention's performance in real‐world situations, efficacy trials determine whether it is beneficial in ideal circumstances. Safety assessments are essential at every trial stage. Phase 3 trials are especially important for identifying less frequent side effects because they involve larger populations (Gartlehner et al. 2010). Treatment recommendations were influenced by the RALES Trial, which showed that spironolactone decreased mortality in patients with severe heart failure by 30%. For Alzheimer's disease, lecanemab has received FDA approval after demonstrating a notable decrease in amyloid beta plaques and a slower rate of cognitive impairment. As demonstrated by the addition of spironolactone to the treatment of heart failure, encouraging trial outcomes may result in revisions to the clinical practice guidelines. To approve new medicines and ensure that they follow the safety and efficacy criteria, regulatory agencies rely on trial data. By being aware of the advantages and disadvantages of various therapies, medical professionals can make well‐informed judgments specific to each patient's needs.

## 5 Conclusions

Plumbagin is a naturally occurring chemical found in the roots of the *Plumbago genus*, particularly *Plumbago zeylanica* and related species. Naphthoquinone has been the focus of numerous studies owing to its potential medical advantages. Current studies are being conducted to evaluate the safety and effectiveness of PB in clinical trials, optimize its formulation and distribution, and obtain a deeper understanding of the mechanisms underlying its activity. Although PB is a bioactive substance with several potential therapeutic applications, further research is necessary to fully comprehend its benefits and drawbacks in clinical settings. PB, which has antimalarial, anti‐obese, antidiabetic, antimicrobial, antiulcer, anti‐inflammatory, antioxidant, and anticancer properties, is found in the plant's roots, leaves, and stem. It has historically been used to treat a wide range of illnesses, such as arthritis, colds, coughs, rheumatic pain, leprosy, anemia, and dysmenorrhea. Owing to its weak solubility, metabolic instability, and transporter efflux, PB has low bioavailability. Despite its potential medicinal benefits, the toxicity of PB restricts its use in clinical settings. Oxidative stress, cytotoxicity, organ toxicity, and genotoxicity, and plumbagin and its derivatives have great therapeutic potential, especially in antibacterial and oncological applications. However, problems such as toxicity and bioavailability must be addressed. There are several intriguing ways to improve the therapeutic efficacy of PB and its derivatives, including combination therapies, chemical modifications, and advancements in drug delivery systems. Further research and clinical studies are required to fully understand the therapeutic potential and ensure human safety.

## Author Contributions

**Souparnika Thekkumkara:** data curation (equal), investigation (equal). **Arenbenla Longchar:** conceptualization (equal), project administration (equal), writing – original draft (equal). **Baskar Venkidasamy:** conceptualization (equal), project administration (equal), writing – original draft (equal). **Benod Kumar Kondapavuluri:** methodology (equal). **Muthu Thiruvengadam:** methodology (equal), writing – original draft (equal). **Mansour Ghorbanpour:** formal analysis (equal), writing – review and editing (equal). **Sathianarayanan Sankaran:** formal analysis (equal), writing – original draft (equal).

## Ethics Statement

The authors have nothing to report.

## Consent

The authors have nothing to report.

## Conflicts of Interest

The authors declare no conflicts of interest.

## Data Availability

No new data were generated or analyzed in this review. Data supporting the findings of this study are available in the referenced works.
